# Novel covalent CDK7 inhibitor potently induces apoptosis in acute myeloid leukemia and synergizes with Venetoclax

**DOI:** 10.1186/s13046-023-02750-w

**Published:** 2023-07-29

**Authors:** Tarang Gaur, Ramulu Poddutoori, Leena Khare, Bhausaheb Bagal, Sonal Rashmi, Nikhil Patkar, Prashant Tembhare, Subramanian PG, Dhanlaxmi Shetty, Amit Dutt, Qi Zhang, Marina Konopleva, Uwe Platzbeckar, Sudeep Gupta, Susanta Samajdar, Murali Ramchandra, Navin Khattry, Syed K. Hasan

**Affiliations:** 1 Hasan Lab, Advanced Centre for Treatment, Research and Education in Cancer (ACTREC), Tata Memorial Centre, Navi, Mumbai, 410210 India; 2 Homi Bhabha National Institute (HBNI), Anushaktinagar, Mumbai, 400094 India; 3 Aurigene Oncology Limited, Electronic City Hosur Road, Bangalore, 560100 India; 4 Department of Medical Oncology, Tata Memorial Hospital, Tata Memorial Centre, Mumbai, 400014 India; 5 Dutt Lab, Advanced Centre for Treatment, Research and Education in Cancer (ACTREC), Tata Memorial Centre, Navi, Mumbai 410210 India; 6 Present Address: CNAG-CRG, Centre for Genomic Regulation (CRG), Barcelona Institute of Science and Technology (BIST), Barcelona, Spain; 7 Hematopathology Laboratory, Advanced Centre for Treatment, Research and Education in Cancer (ACTREC), Tata Memorial Centre, Navi, Mumbai, 410210 India; 8 Department of Cytogenetics, Advanced Centre for Treatment, Research and Education in Cancer (ACTREC), Tata Memorial Centre, Navi, Mumbai, 410210 India; 9 Department of Leukemia, The University of Texas MD Anderson Cancer Center, Houston, TX 77030 USA; 10 Albert Einstein College of Medicine, Bronx, NY 10461 USA; 11 Medical Clinic and Policlinic I, Hematology and Cellular Therapy, University Hospital Leipzig, Johannisallee 32, 04103 Leipzig, Germany

**Keywords:** Acute myeloid leukemia, Venetoclax resistance, CDK7 inhibition, CRISPR/Cas9, Xenografts, Cell cycle, Apoptosis

## Abstract

**Introduction:**

The emergence of resistance to the highly successful BCL2-directed therapy is a major unmet need in acute myeloid leukemia (AML), an aggressive malignancy with poor survival rates. Towards identifying therapeutic options for AML patients who progress on BCL2-directed therapy, we studied a clinical-stage CDK7 inhibitor XL102, which is being evaluated in solid tumors (NCT04726332).

**Materials and methods:**

To determine the anti-proliferative effects of XL102, we performed experiments including time-resolved fluorescence resonance energy transfer, target occupancy, cell cycle and apoptosis-based assays. We also included genetically characterized primary myeloid blasts from de novo and relapsed/refractory AML patients. For mechanistic studies, CRISPR/Cas9 mediated knockout of CDK7 and c-Myc and immunoblotting were performed. NOD/SCID orthotropic and subcutaneous AML xenografts were used to determine anti-leukemic effects. To assess the synergistic effects of XL102 with Venetoclax, we performed RNA sequencing and gene set enrichment analysis using Venetoclax sensitive and resistant model systems.

**Results:**

XL102, a highly specific, orally bioavailable covalent inhibitor of CDK7. Inhibitory effect on CDK7 by XL102 in primary myeloid blasts (*n* = 54) was in nanomolar range (mean = 300 nM; range = 4.0-952 nM). XL102 treated AML cells showed a reduction in phosphorylation levels of Serine 2/5/7 at carboxy-terminal domain of RNA polymerase II. T-loop phosphorylation of CDK1(Thr161) and CDK2(Thr160) was inhibited by XL102 in dose-dependent manner leading to cell-cycle arrest. c-Myc downregulation and enhanced levels of p53 and p21 in XL102 treated cells were observed. Increased levels of p21 and activation of p53 by XL102 were mimicked by genetic ablation of CDK7, which supports that the observed effects of XL102 are due to CDK7 inhibition. XL102 treated AML xenografts showed remarkable reduction in hCD45 + marrow cells (mean = 0.60%; range = 0.04%-3.53%) compared to vehicle control (mean = 38.2%; range = 10.1%-78%), with corresponding increase in p53, p21 and decrease in c-Myc levels. The data suggests XL102 induces apoptosis in AML cells via CDK7/c-Myc/p53 axis. RNA-sequencing from paired Venetoclax-sensitive and Venetoclax-resistant cells treated with XL102 showed downregulation of genes involved in proliferation and apoptosis.

**Conclusion:**

Taken together, XL102 with Venetoclax led to synergistic effects in overcoming resistance and provided a strong rationale for clinical evaluation of XL102 as a single agent and in combination with Venetoclax.

**Supplementary Information:**

The online version contains supplementary material available at https://doi.org/10.1186/s13046-023-02750-w.

## Key points

• XL102 is a novel, highly specific, orally bioavailable covalent inhibitor of CDK7

• CDK7 inhibition induces cell cycle arrest and apoptosis via CDK7/c-Myc/p53 axis in AML

• XL102 synergizes with Venetoclax in AML

**Supplementary Information:**

The online version contains supplementary material available at https://doi.org/10.1186/s13046-023-02750-w.

## Introduction

Acute Myeloid leukemia (AML) is a biologically heterogeneous aggressive malignancy of the hematopoietic system with poor survival rates. Improved understanding of AML biology and impressive translational development programs, both as a frontline and in relapsed/refractory settings, led to multiple new therapeutic options for patients with AML. Despite these translational developments, most patients still succumb to the disease. Therefore, additional therapeutic options and treatment combinations that can be more specific and less toxic are urgently needed. Recent advances in biology and drug development are rapidly transforming the therapeutic landscape of AML. During the past five years, the US Food and Drug Administration (FDA) fast-track approval of 10 new-targeted agents indicates that AML treatment is moving beyond conventional cytotoxic agents in the frontline and relapsed/refractory settings [1–5]. Of these newly approved agents, the majority are used in specific biological subsets of patient populations, including patients who are unfit for intensive chemotherapy [5, 6]. Harnessing insights from the AML biology, novel therapeutic modalities such as inhibition of cyclin-dependent kinases and B-cell lymphoma 2 (BCL2) proteins are emerging wherein therapeutic strategies can be reframed regardless of patient fitness or specific biological subgroup harboring genetic mutations [7–9]. However, resistance to BCL2 directed therapy emerges as major unmet need in AML.

The cyclin-dependent kinase (CDK)7 is responsible for phosphorylation of serine 5 (Ser5), serine 7 (Ser7), and serine 2 (Ser2) of the conserved regions of carboxy-terminal domain (CTD) of RNA polymerase II (RP Pol II), leading to transcriptional initiation and elongation. The covalent inhibitors of CDK7 have been emerging fast, but only a couple of them could enter clinical trials. These inhibitors covalently bind to CDK7 outside of the kinase domain and have shown excellent anti-tumor activity by exploiting the transcriptional vulnerability of oncogenes and cell cycle proteins in diverse cancer types [10–15].

In an effort to identify therapeutic options for AML patients who progress on BCL2 directed therapy, we evaluated a clinical-stage CDK7 inhibitor XL102, which is being evaluated in solid tumors (NCT04726332). XL102, a highly specific, orally bioavailable covalent inhibitor of CDK7. Our findings from the biochemical and cell-based analyses indicate the potency and selectivity of CDK7 inhibition by XL102. Furthermore, XL102 inhibits phosphorylation levels of Ser 2/5/7 at carboxy-terminal domain of RNA polymerase II, T-loop phosphorylation of CDK1(Thr161) and CDK2(Thr160) in dose-dependent manner leading to cell-cycle arrest in AML cell lines and primary blasts. Anti-proliferative activity correlates with the downregulation of c-Myc resulting in the upregulation of p53-mediated signaling leading to cell cycle arrest and programmed cell death. The transcriptional targets of XL102 were identified using paired Venetoclax sensitive/ resistant model by RNA sequencing. Our data provide novel insights into the mechanisms of CDK7 inhibition via CDK7/c-Myc/p53 axis that has not been reported previously in solid or liquid tumors and combined treatment with XL102 and Venetoclax led to synergistic effects in overcoming resistance to Venetoclax in AML.

## Materials and methods

### Cell lines and inhibitors

AML cell lines were procured from the European Institute of Oncology (Milan, Italy). Professor Marina Konopleva gifted paired Venetoclax resistant and sensitive MOLM13 cells from MD Anderson, Houston USA [16]. MOLM13 and MV4-11 cells were cultured in the recommended media containing 10% fetal bovine serum (FBS) and 10 mM L-glutamine (Gibco, Thermo Fisher Scientific US) whereas OCI AML2 and OCI AML3 cells were cultured in 20% alpha MEM (Gibco, Thermo Fisher Scientific US) and maintained at 37 °C with 5%CO_2_. XL102 was developed in collaboration with the Aurigene Oncology Limited, Bangalore. Venetoclax and Cytarabine were purchased from Sellekchem, US.

### Potency analysis of XL102

The inhibitory activity of the test compound was assessed by the LANCE time-resolved fluorescence resonance energy transfer (TR-FRET) assay which detects the ATP-dependent phosphorylation of U-Light-myelin basic protein (MBP) substrate peptide by CDK7 was performed in 384-well plate format. Briefly, the enzyme reaction was run in reaction buffer (20 mM HEPES (pH 7.5), 10 mM MgCl2, 0.01% Triton X100, 100 μM Sodium Orthovanadate, 1 mM DTT) with the final concentration of the ATP substrate at 1 mM, U-Light-MBP substrate peptide at 100 nM, and CDK7 at 10 nM. After the pre-incubation of the compound and enzyme for 60 min at room temperature, the kinase reaction was initiated by the addition of ATP and the peptide substrate. The reaction was terminated by the addition of 40 mM EDTA and 1 nM Eu-labeled anti-phospho-MBP-binding protein antibody after the 20, 60, 180 min incubation of kinase reaction at room temperature.

### Target occupancy assay

MOLM13 cells were treated with 0.1, 0.5, 1.0 and 2.0 μM concentrations of the XL102 for 3 h. In brief, 20 million cells were harvested at 0 h, 6 h, 16 h and 24 h post wash out, using cell lysis buffer. BioTHZ1 (1 μM) in DMSO was added to 1 mg of the protein lysate and the samples were transferred to a plate shaker for incubation overnight at 4 °C. On the following day, BioTHZ1 containing samples were added to Streptavidin beads and incubated overnight before performing pull down assay. For total CDK7 Western blot samples for each treatment were prepared using 30 μg protein of each lysate in 1X Protein loading dye. For Streptavidin pull down samples, the tubes were washed with 1 mL of TBST five times by centrifugation at 2000 rpm for 1 min and discarding the supernatant every time. For pulldown samples 30 μL of 1X Protein loading dye was added. The total CDK7 and pulldown samples were resolved on this gel and transferred to PVDF membranes (Merck Millipore, Massachusetts, US). The PVDF membranes were blocked with LI-COR blocking buffer (LI-COR Biosciences, Nebraska, US) for 60 min and then incubated with primary antibody at 4 °C overnight. Antibodies used against various proteins were CDK7 (Bethyl Laboratories, Massachusetts, US) and Beta actin (Santa Cruz Biotechnology, Texas, US). Thereafter, the PVDF membranes were washed three times for 30 min and incubated with respective IRDye tagged secondary antibodies (Licor Biosciences) for 60 min at room temperature. Following incubation with IRDye tagged secondary antibodies (LI-COR Biosciences, Nebraska, US), membranes were imaged on an Odyssey CLx Imager (LI-COR Biosciences, Nebraska, US). For each sample, band intensities of CDK7 pulled down by streptavidin beads and total CDK7 from the input sample were estimated from the raw data image files using Image studio software and exported to an excel sheet. The percentage of CDK7 occupancy was calculated for compound treatment samples with reference to the untreated samples. The untreated sample was considered to have occupancy of 100%.

### Primary AML samples, cytogenetic analyses and mutation detection by NGS

The peripheral blood/bone marrow samples from de novo AML patients who achieved complete remission (*n* = 42) and relapsed/refractory (*n* = 12) were collected and processed for the isolation of myeloid blasts for functional assays and DNA/RNA for molecular analysis. Further to evaluate the selective toxicity of XL102, peripheral blood mononuclear cells (PBMCs) of healthy individuals (*n* = 15) were collected from blood bank. All patients and donors provided written informed consent following the Declaration of Helsinki, and the ethics Committee of Tata Memorial Centre, Mumbai (TMC-IEC III) DCGI registration number:IECIII: ECR/149/Inst/MH/2013 approved the study (approval reference number: 900516/2018). All AML patients included in this study underwent Cytogenetic analyses [17] by standard techniques of chromosome banding and FISH; mutation profiling using in-house developed hybrid capture oligonucleotide probes based next generation sequencing (NGS) assay [18]. The clinico-biological features of patients are presented in Table 1.

**Table 1:** Clinico-biological features of 54 AML patients included in the study

**UPN**^a^	**XL102 IC**_**50**_** (μM)**	***de novo*/Relapsed Refractory**	**Age/ Gender**	**%Blasts (BM)**^b^	**Karyotyping at diagnosis**	**FISH at diagnosis**	**Mutations identified**
1	0.22	de novo	34/M	58	ND	Tri-tetrasomy of chromosome 21/3–4 copies of RUNX1 allele in 90% cells	Monoallelic CEBPA
2	0.31	de novo	53/M	40	ND	inv(16)/t(16;16) in 90% cells	Not detected
3	0.95	de novo	22/M	92	ND	No abnormalities detected	FLT3-ITD
4	0.49	de novo	29/M	88	46,XY,t(9;11)(p21;q23)[12]/ 46,XY[13]	Partial deletion of MLL in 92% cells (Positive for KMT2A rearrangement:t(9;11)(p21;q23))	NRAS c.35G > A
5	0.03	de novo	37/M	55	ND	RUNX1-RUNX1T1 fusion: t(8;21) in 98% cells	NRAS c.34G > T
6	0.34	Relapsed	45/M	99	ND	No abnormalities detected	Biallelic CEBPA
7	0.02	de novo	27/F	38	46,XX[15]	No abnormalities detected	Not detected
8	0.02	de novo	35/F	75	46,XX[20]	No abnormalities detected	FLT3-ITD
9	0.18	Refractory	49/F	59	ND	MLL rearrangement in 94% cells	Not detected
10	0.26	de novo	25/M	96	46,XY[20]	3 copies of RUNX1 allele/ trisomy 21 in 95% cells and 3 copies of RUNX1T1/ trisomy 8 in 15% cells	Biallelic CEBPA
11	0.08	de novo	61/F	42	ND	No abnormalities detected	Not done
12	0.01	Refractory	23/F	78	ND	No abnormalities detected	Not done
13	0.12	de novo	41/F	82	ND	del(5q) in 94% cells, tri-tetrasomy of 11 in 80% cells and TP53 deletion in 90% cells	Not done
14	0.47	de novo	23/M	75	45,X,-Y,t(8;21)(q22;q22)[10]	RUNX1-RUNX1T1fusion: t(8;21) in 90% cells	Not done
15	0.31	Refractory	39/M	80	ND	No abnormalities detected	Not done
16	0.14	de novo	49/F	48	46,XX[11]	No abnormalities detected	ASXL1 c.2113G > T, EZH2 c.1562G > A
17	0.16	de novo	45/F	40	46,XX[12]	No abnormalities detected	NPM1 Type A, IDH1 c.394C > T, NRAS c.99 T > A
18	0.21	de novo	23/M	65	ND	No abnormalities detected	WT1 c.1154_1155insACTCTTGTAG, RAD21 c.374dup, NPM1 Type A, TET2 c.3646C > T, FLT3 c.2503G > T
19	0.44	de novo	22/F	90	ND	RUNX1-RUNX1T1 fusion: t(8;21) in 98% cells	NRAS c.38G > A, CSF3R c.1853C > T
20	0.58	Relapsed	39/M	70	ND	No abnormalities detected	IDH2 c.515G > A, RUNX1 c.808_811dup4
21	0.10	de novo	35/M	45	49,XY, + 6, + 8,inv(16)(p13.3q22), + 21[7]/48,XY, + 4, + 8,inv(16))(p13.3q22)[3]/46,XY[1]	CBFB rearrangement: inv(16)/t(16;16) in 90% cells, trisomy 8 and trisomy 21 in 90% cells	KIT c.2446G > T, NRAS c.182A > G
22	0.35	de novo	49/F	64	46,XX[19]	No abnormalities detected	NPM1 Type A, IDH1 c.395G > A, NRAS c.38G > A
23	0.008	de novo	35/F	85	ND	No abnormalities detected	NPM1 Type B, FLT3-ITD,
24	0.30	de novo	38/F	73	ND	No abnormalities detected	EZH2 c.1846G > A, SMC1A c.287G > A, IDH1 c.394C > T, NRAS c.35G > A
25	0.79	de novo	48/M	57	45,X,-Y,t(8;21)(q22;q22)[5]/46,XY[9]	RUNX1/RUNX1TI fusion: t(8;21)(q22;q22) in 95% cells	KIT c.2466 T > G, KIT c.1252_1253insCTTTCT,
26	0.25	de novo	56/M	70	ND	No abnormalities detected	NPM1 c.858_859insTCTG, FLT3-ITD, IDH2 c.419G > A
27	0.49	de novo	34/M	81	ND	RUNX1/RUNX1TI fusion: t(8;21)(q22;q22) in 96% cells	KIT c.2466 T > G (32.12%)
28	0.69	de novo	45/F	85	46,XX[20]	No abnormalities detected	NPM1 Type A, DNMT3A c.2172C > A, IDH2 c.419G > A, FLT3 c.2506_2508delATC, KRAS c.35G > T
29	0.60	de novo	41/F	70	46,XX[20]	No abnormalities detected	NPM1 Type A, IDH1 c.394C > A, NRAS c.181C > A, TET2 c.3583A > G
30	0.51	Refractory	34/M	89	46,XY[25]	del(7q) in 10% cells. 3 copies of MLL allele due to structural rearrangement of 11q region in 15% cells	DNMT3A c.2645G > A, IDH1 c.394C > T, NRAS c.35G > A
31	0.24	de novo	20/M	85	47,XY, + 4,t(8;21)(q22;q22)[5]/46,XY,t(8;21)(q22;q22)[9]/46,XY[1]	RUNX1/RUNX1TI fusion: t(8;21)(q21.3;q22) in 90% cells	KIT c.2447A > T
32	0.56	de novo	32/F	96	ND	No abnormalities detected	NPM1 Type A, TET2 c.3461G > A, FLT3-ITD
33	0.12	de novo	47/F	32	ND	No abnormalities detected	Not done
34	0.05	de novo	40/M	74	ND	No abnormalities detected	Not done
35	0.84	de novo	26/F	69	ND	No abnormalities detected	Not done
36	0.36	Refractory	38/F	92	ND	Monosomy 7 in 97% cells	Not detected
37	0.92	de novo	22/M	35	46,XY [19]	No abnormalities detected	Not detected
38	0.13	Refractory	44/F	80	ND	No abnormalities detected	Not done
39	0.20	Refractory	33/F	68	46,XX,t(8;21)(q22;q22)[9]/46,XX[2]	RUNX1-RUNX1T1 fusion: t(8;21) in 94% cells	FLT3-ITD
40	0.33	Relapsed	31/M	60	46,XY [14]. Abnormal clone not proliferated	RUNX1-RUNX1T1 fusion: t(8;21) in 96% cells	Not detected
41	0.16	de novo	52/F	60	ND	RUNX1-RUNX1T1fusion: t(8;21) in 96% cells	Not done
42	0.86	de novo	36/M	70	46,XY[20]	No abnormalities detected	RUNX1 c.849dupT, IDH1 c.394C > T, DNMT3A c.2644C > T, TET2 c.4210C > T,SF3B1 c.1078A > G
43	0.004	Relapsed	46/M	74	46,XY [15]. Abnormal clone not proliferated	RUNX1-RUNX1T1 fusion: t(8;21) in 94% cells	Not detected
44	0.36	de novo	33/F	89	ND	No abnormalities detected	Not done
45	0.31	de novo	24/M	83	47,XY, + 8[18]/46,XY[2]	Trisomy in 85% of cells	FLT3-ITD
46	0.61	Refractory	39/M	66	46,XY,der(18;21)(p10;q10) + 21[30]	Trisomy 21 in 92% cells	Biallelic CEBPA, GATA2 c.953C > G
47	0.83	de novo	61/M	63	47,XY, + 11[6]/46,XY[14] []	Trisomy 11 in 50% cells	Not done
48	0.6	de novo	45/M	94	44,X,-Y,-7[14]/45,XY,-7[2]/46,XY[1]	Monosomy 7 in 95% cells	KRAS c.35G > C, SF3B1 c.2098A > G
49	0.05	de novo	24/M	85	ND	Trisomy 8 in 85% of cells	NPM1 type A; FLT3-ITD
50	0.07	de novo	45/M	68	46,XY [15]	No abnormalities detected	DNMT3A c.2644C > T, IDH2 c.515G > A, RUNX1 c.485G > A
51	0.07	de novo	46/F	55	46,XX [20]	Deletion(5q) in 70% cells	Not done
52	0.10	de novo	40/F	70	46,XX[11]	No abnormalities detected	NPM1 type A
53	0.15	de novo	35/M	90	ND	No abnormalities detected	Not done
54	0.09	de novo	56/M	86	46,XY[13]	No abnormalities detected	Not done

*ND* Not Done (Conventional karyotyping was not possible due to absence of metaphases)

^a^UPN Unique Patient Number

^b^BM Bone marrow

### Antiproliferation and combination index (CI) analysis

Enrichment of leukemic blasts from blood/marrow was carried out using indirect microbeads based on the magnetic-activated cell sorting using cocktail containing CD3, CD20, CD16, CD14 for negative selection (Miltenyi Biotec, US). The purity of the MACS enriched CD34 + cells from healthy donor mobilised peripheral blood was assessed by flow cytometery and was consistently > 95%. Cytotoxicity was assessed using CellTiter-Glo (CTG) assay. Starting from 10 μM with three fold serial dilutions to 0.04 μM (10, 3.33,1.11,0.37,0.123,0.04 μM) in triplicate wells for 72 h. The CTG assay was performed according to the manufacturers’ instructions (Promega, Madison, US). Cell viability at each drug concentration was calculated, and the dose–response curve was generated using GraphPad Prism. CompuSyn software (ComboSyn, Inc) was used to determine the synergism of drug combination in the cell lines and patient samples. The software was based on the Chou-Talalay method [19] for drug combination. The Combination index values obtained provides a quantitative measure for additive effect (CI = 1), synergism (CI < 1) or antagonism (CI > 1) in drug combinations.

### Cell cycle, CFSE (Carboxyfluorescein succinimidyl ester) staining and apoptosis assays

Cell-cycle analysis was performed 24 h after XL102 treatment using flow cytometry. AML cells were fixed in chilled ethanol and incubated with propidium iodide (PI) and RNase Solution (Cell Signaling Technology, US) for 30 min at room temperature. Cells were labeled with CFSE stain (2 μM) and incubated for 15 min at 37 °C. The cells were washed and seeded in 6 well plates for 24 h of XL102 treatment. As the cells divide, the fluorescence intensity of the dye decreases, and the degree of cell proliferation was assessed by flow cytometry. The population of living cells was gated, and 50,000 cells for each condition were randomly selected and plotted. The geometric mean for each population was compared between cells alone and treated with XL102. For the synchronization experiment, cells were subjected to serum starvation for 24 h to arrest them in the G0/G1 phase, followed by the addition of mimosine (40 μM) to the medium to synchronize the cells in the G1 phase and then release them from synchronization by adding serum back to the medium before treating with XL102. This allows the cells to progress through the cell cycle in a more synchronous manner. Apoptosis was determined using the Dead Cell Apoptosis Kit with Annexin V-FITC and PI (ThermoFisher, US). Flow cytometric analysis was done on a FACS Attune cytometer and data were analyzed using Modfit (Maine, US) and FlowJo software (Orlando, US).

### Cell lysate preparation and Western blot assays

For protein analysis, cells were washed with 1X phosphate buffered saline (PBS) followed by lysis with RIPA buffer (50 mM Tris, pH 7.5, 150 mM NaCl, 1% NP-40, 0.5% sodium deoxycholate, and 0.1% SDS) supplemented with protease inhibitor (Roche Diagnostics, Mannheim Germany). Protein concentrations were determined by using the Bradford reagent (Sigma Aldrich, US). 30 µg of protein was resolved on 6–10% SDS-PAGE and subsequently transferred onto nitrocellulose membrane (Biorad, California US). The nitrocellulose membrane was blocked with 5% BSA and incubated with primary antibodies overnight at 4 °C with gentle mixing. Next day, after 3 TBST washes, the membranes were incubated with secondary anti-rabbit/mouse for 1 h at room temperature. The signal was visualized using enhanced chemiluminescence western blot analysis system (Bio-Rad, California US). All experiments were done in triplicates. Antibodies used were RNA Pol II, #2629, p-RNA pol II ser2 #8798, p-RNA pol II Ser5, #13523, p-RNA pol II ser7, #13780 p-CDK2 (Thr170) #2561, CDK2#2546, Myc #5605, p53 #9282, p21 #2947, p27 #3686, Cleaved PARP #5625, XIAP #2042, MCL1 #4572, BCL2 #2876, BCL-XL #2762, cleaved-caspase3 #9661, (# catalogue number; Cell Signaling Technologies, Massachusetts, US) p-CDK1 (Thr 161) (Thermo Fisher PA5-117191).

### Knockout experiments using CRISPR/Cas9 plasmids and transduction

#### sg_c-Myc

The c-Myc knockout (c-Myc^KO^) was performed using LentiCRISPR v2 plasmid purchased from Addgene (Massachusetts, US). To generate lentiviral single guide RNA (sgRNA) plasmid, guide RNA targeting c-Myc were annealed and cloned into the LentiCRISPR plasmid. The gRNA sequences for c-Myc are: Fwd:5’-CACCGGCCGTATTTCTACTGCGACG-3’; Rev:5’-ACCGTCGCAGTAGAAATACGGCC-3’. Viral particles were generated by transfection of HEK293T cells with carrier plasmid along with packaging and envelope vectors pMD2.G and psPAX, respectively. After 48 h, supernatant media containing viral particles were harvested, filtered and transduced with MOLM13 cells, followed by puromycin selection at 0.5 μg/mL [20].

#### sg_CDK7

The CDK7 knockout (CDK7^KO^) was done using Edit-R All-in-one lentiviral system (Dharmacon, Horizon Discovery, UK). The CDK7 target sequence was: 5’-CTTAATGGCGACAATTTGGT-3’. The viral particles targeting CDK7 were directly transduced with MOLM13 cells followed by 1 week of puromycin selection at 0.5 μg/mL. After puromycin selection for both sg_c-Myc and sg_CDK7, we performed single-cell cloning by limiting dilution keeping 100 cells in 10 mL in 96 well plate. The puromycin positive cells were allowed to grow for 4 weeks to generate clones, and target gene knockout was validated by immunoblotting.

### Subcutaneous and orthotopic xenografts of AML

All NOD/SCID mice experiments were conducted in accordance with guidelines approved by animal ethics committee of Tata Memorial Centre, Mumbai (study reference approval number IAEC/13/2018). MOLM13 cells were subcutaneously injected into the 6–8 weeks old NOD/SCID mice. The animals were routinely weighed and tumors were measured thrice weekly. Tumor volumes were calculated using the formula 1/2[length (mm)]x[width (mm)]^2^. After 10 days, when tumor sizes reached approximately 150–200 mm^3^, mice were divided into three groups of 9 mice in each group. The control group (0.1% DMSO), XL102 group (60 mg/Kg/oral daily), and Cytarabine group (20 mg/Kg/ intraperitoneal daily). To assess the in vivo synergy between XL102 and Venetoclax, MOLM13 Venetoclax-resistant cells were injected in NOD/SCID mice. After tumor formation, animals were randomized in four groups and treated with vehicle control, XL102, Venetoclax and a combination of Venetoclax and XL102. Mice were then sacrificed, and tumor specimens of mice were decalcified and paraffin-embedded. The slides were stained with hematoxylin and eosin (H&E) for assessment of tumor necrosis and fibrosis.

For orthotopic AML xenografts, 2 million MOLM13 cells were injected by tail vein after 24 h of busulfan (20 mg/kg) based conditioning regimen. The AML engraftment was confirmed using blood collected by retro-orbital bleeding followed by staining with anti-human (hCD45) antibody and analysis through flow cytometry. The animals were divided into three groups similar to the subcutaneous experiment followed by 12 days of treatment. After 12 days of treatment, mice were sacrificed. The bone marrow was flushed in PBS and made into single cell suspension followed by staining with anti-mouse (mCD45) antibody and hCD45 for analysis by flow cytometry. The spleen of animals from all three groups was fixed in 10% formalin solution (Sigma-Aldrich, US) and processed by the Histology Core of ACTREC.

### Immunohistochemistry (IHC)

IHC was done using formalin-fixed, paraffin-embedded 5-mm-thick tumor or spleen section. The tissues were dewaxed by Xylene and rehydrated in consecutive ethanol baths. Standard Mayer’s hematoxylin and eosin staining was performed. Sodium citrate antigen retrieval buffer was used and sections were stained with anti Ki67 (Abcam Cambridge, UK) and cleaved caspase3 antibodies (Cell Signaling Technologies, Massachusetts, US).

### RNA Sequencing and real-time quantitative PCR (RQ-PCR)

We have labeled Venetoclax-sensitive cells with XL102 treatment as Ven^S^T while untreated cells were marked as Ven^S^U. Similarly, Venetoclax-resistant cells with and without XL102 treatment were labeled as Ven^R^T and Ven^R^U respectively. RNA extracted from these cells was quantified using the Qubit RNA HS assay kit (Thermo Fisher Scientific) and diluted to 100 ng/μl. The quality of total RNA was confirmed by Bioanalyzer. Samples with RNA integrity numbers ≥ 8 were selected for RNA library preparation in an ISO/IEC 17025-accredited protocol (TruSeq RNA library preparation kit v2, Illumina, San Diego, CA). One μg of total RNA was isolated from paired Venetoclax sensitive and resistant MOLM13 cells with and without XL102 treatment, followed by DNAse I and silica-membrane purification (RNeasy kit, Qiagen, Germany).

After enrichment of mRNA by oligo dT magnetic beads and fragmentation, cDNA synthesis was performed in duplicate for each sample, followed by adapter ligation and PCR amplification. The obtained libraries were pooled and diluted to final optimal loading concentration before cluster amplification on the Illumina flow cell. Once the cluster generation is completed, the clustered flow cell is loaded on Illumina HiSeq X instrument to generate 167million, 150 bp paired end reads per sample. Image analysis, base calling, and quality check was performed with Illumina data analysis pipeline RTA v1.18.64. RNAseq reads were provided in compressed Sanger FASTQ format. For realtime PCR based validation, RNA extraction from leukemia cells, untreated and treated with XL102 was carried out by Trizol based method, cDNA synthesis with high capacity cDNA synthesis kit (Thermo Fisher Scientific, USA) and PCR using LightCycler 96 Real-Time PCR System (Roche Diagnostics GmbH, Germany). Primer sequences used for RQ-PCR validation has been added in Supplementary Table 1.

### Statistical analysis

GraphPad Prism version 4.00 (GraphPad Software, San Diego, CA) was used for statistical analysis. Values between groups were compared using one-way analysis of variance (ANOVA), nonparametric test. Unpaired t-tests were performed to test differences in vehicle and compound treated groups. Kaplan–Meier method was used to estimate the survival, and group comparisons were performed using the log-rank test. The mean ± SD is graphed. *P* values < 0.05 were considered significant, where *p* value < 0.05 (^∗^), *p* value < 0.01(^∗∗^), *p* value < 0.001 (^∗∗∗^), *p* value < 0.0001 (^∗∗∗∗^).

## Results

### Potency and CDK7 dependent phosphorylation inhibition by XL102 in AML cells

**Fig. 1:**
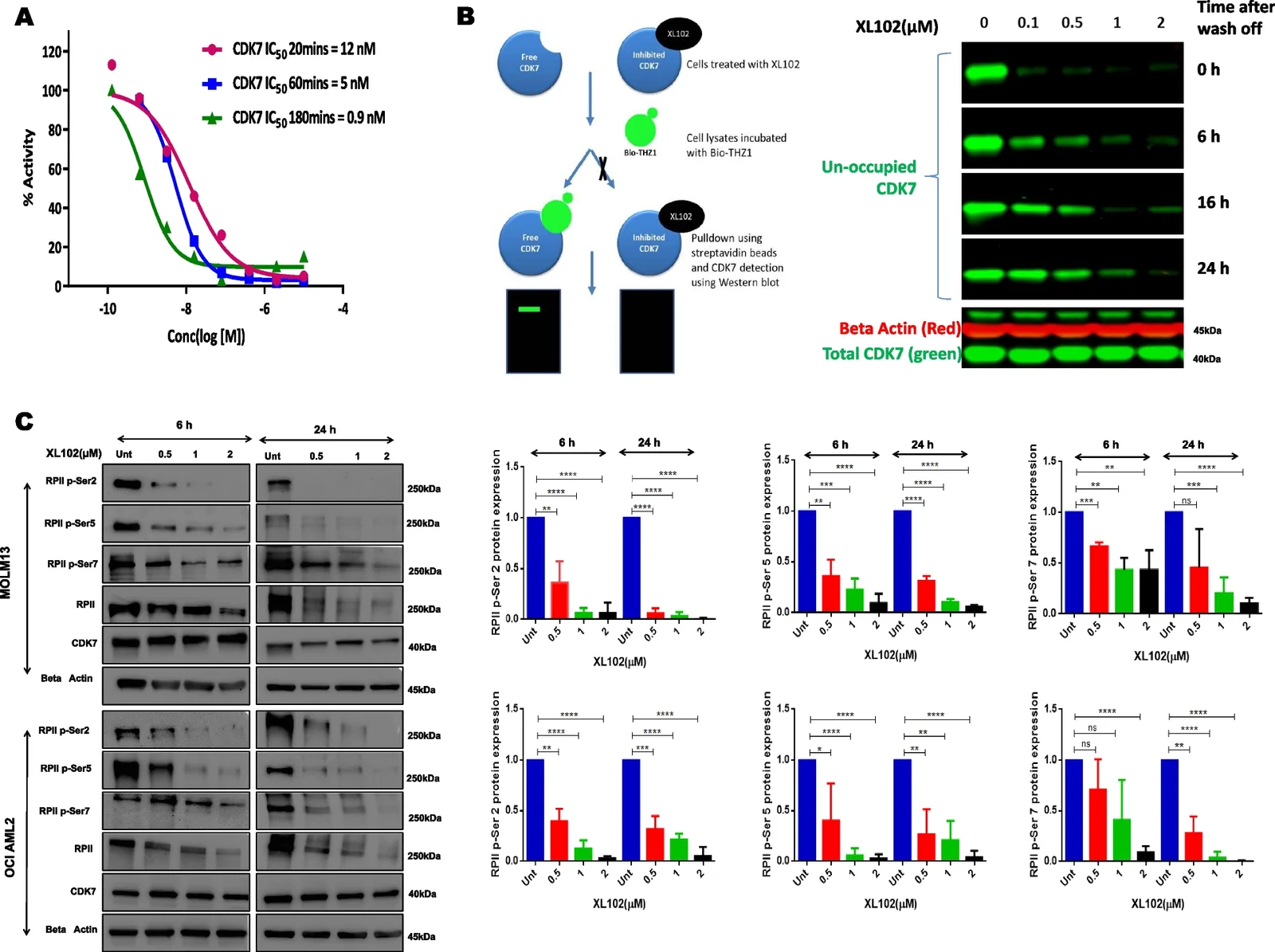
Efficacy of XL102 and its effects on downstream targets of CDK7. **A** Kinase activity of CDK7 in the presence of XL102 was measured using the LANCE TR-FRET in vitro kinase assay by incubating both CDK7 and XL102 followed by addition of ATP and U-light-MBP peptide substrate. Time-resolved fluorescence (excitation, 320 nm; emission donor, 615 nm; emission acceptor, 665 nm) was monitored by using 2030 multilabel reader Victor5 (PerkinElmer). The IC_50_ values were derived by fitting a sigmoidal dose–response curve to a plot of assay readout over inhibitor concentration, computed with the Graph Pad Prism. **B** The pull-down assay using bio-THZ1 show a dose dependent target engagement in AML cells harvested after 3 h of XL102 treatment followed by washing to remove any unbound XL102 (time point-0 h). **C** XL102 inhibited CTD phosphorylation of conserved residues of RPII in AML cells in dose dependent manner at 6 h and 24 h along with quantification of Western blot data. Results are the mean ± SD of three independent experiments

Biochemical evaluation of XL102 using the LANCE TR-FRET in vitro kinase assay indicated the potency of XL102 shifts over time due to its attachment to CDK7. The target-dependent inhibition (IC_50_) of CDK7 by XL102 at 20, 60 and 180 min was found to be 12 nM, 5 nM and 0.9 nM, respectively (Fig. 1A). We observed that CDK7 engagement was started at 0.1 μM of XL102 and became significantly engaged at higher concentrations (Fig. 1B). However, when cells were harvested after different time points (6 h, 16 h and 24 h) of XL102 wash-off, newly synthesized CDK7 protein reappeared (Fig. 1B). Consistent with the reported role of CDK7 inhibitors in transcriptional control, XL102 led to concentration dependent decrease in phosphorylation of RP II of CTD domain at Ser2, Ser5, and Ser7 in two AML cell lines in time-dependent manner (Fig. 1C). We also observed XL102 decreases RP II levels in MOLM13 and OCI AML2 cells. As previously reported CDK7 activity is critical for properly maintaining basal levels of RP II and producing stable transcripts. However perturbation of this activity by XL102 can cause a decrease in basal levels of RP II by inhibiting its phosphorylation [21–23]. The quantification graphs of basal levels of RP II and CDK7 are provided in Supplementary Fig. 1.

### Antiproliferative effect of XL102 in AML cell lines and patient-derived myeloid blasts leading to apoptosis

**Fig. 2:**
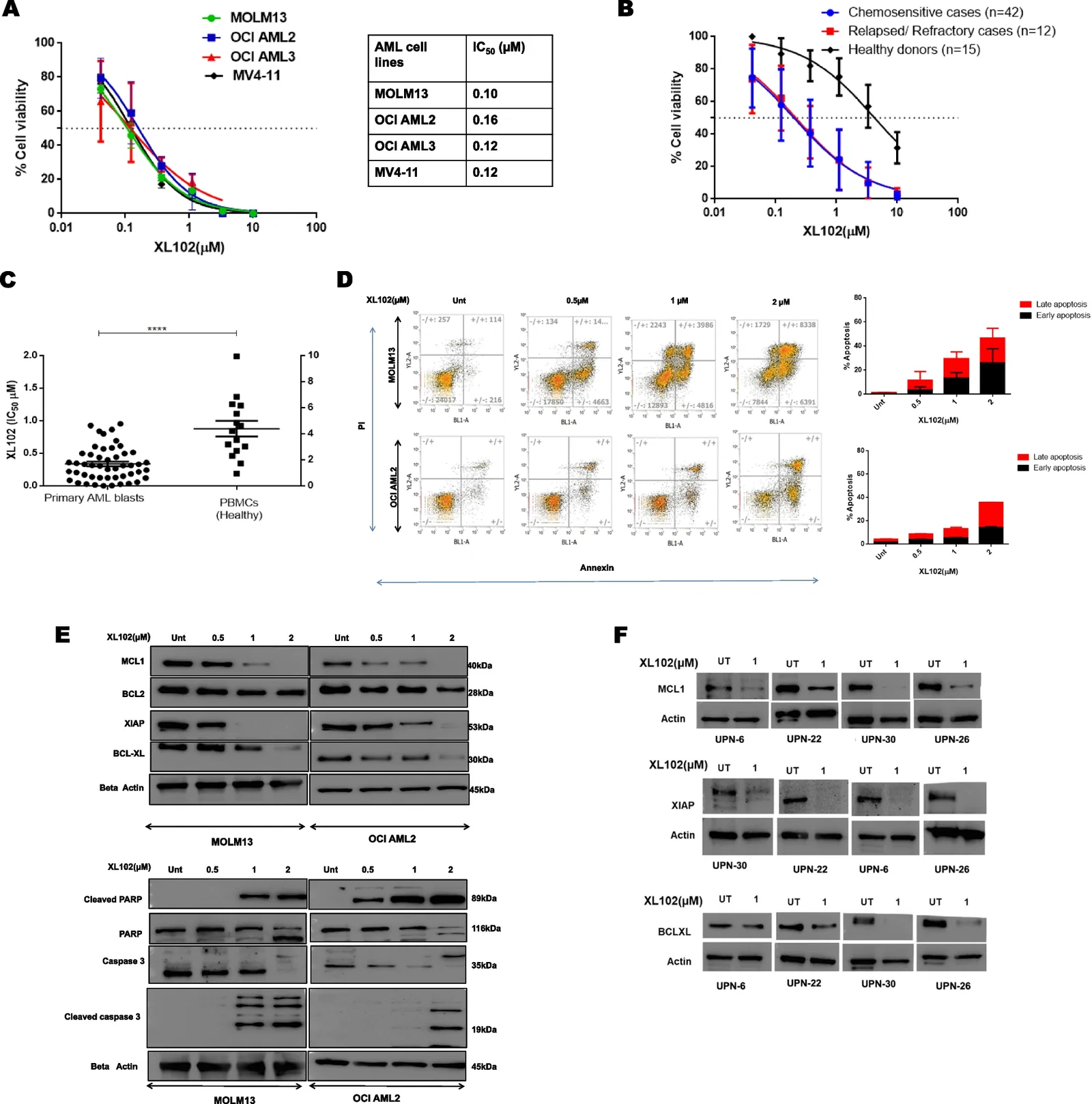
Anti-leukemic activity of XL102 in AML cell lines, primary myeloid blasts and healthy individuals. **A** The antiproliferative effect of XL102 using different AML cells was determined using CTG assay after 72 h of drug treatment. Values were calculated using GraphPad Prism by log-transforming the data and fitting it to a nonlinear regression. **B** Antiproliferative analysis of XL102 in patient derived myeloid blasts of de novo (*n* = 42), relapsed/refractory (*n* = 12).and mononuclear cells derived from healthy individuals (*n* = 15) **C** Comparative analyses of XL102 cytotoxicity in AML blast and healthy PBMC. **D** Cell apoptosis was measured using a flow cytometry analysis of Annexin/PI staining after 24 h of XL102 treatment at the indicated concentrations in MOLM13 and OCI AML2 cells **E** Change in expression of antiapoptotic protein after 24 h of drug treatment in AML cells. Induction of cleaved PARP and cleaved Caspase-3 in dose dependent manner. **F** Change in expression of antiapoptotic protein in patient derived blast after 24 h of drug treatment. All the western blots are performed in triplicates

Despite the genetically heterogeneous nature of AML cell lines, the inhibitory concentration of XL102 was found to be in nanomolar range (100-200 nM) (Fig. 2A). The viability data of myeloid blasts from primary and relapsed/refractory AML patients revealed the mean IC_50_ of XL102 was 0.33 μM (range 0.008–0.95 μM) and 0.30 μM (0.004–0.61 μM) respectively. The individual IC_50_ data, and the detailed baseline clinical-biological features of 54 AML patients have been provided in Table 1. The error-corrected NGS data of 51 genes was available in 39 out of 54 cases. Based on the mean IC_50_ (0.32 μM), mutational data of most commonly mutated FLT3-ITD (8/39) was analyzed by dividing patients into IC_50_ ≤ 0.32 (*n* = 21) and > 0.32 μM (*n* = 18). We did not find any significant association between FLT3-ITD identified in patients and IC_50_ values (*p* = 0.43). This suggests that antiproliferative activity of XL102 is independent to mutational status of AML patients. The mean IC_50_ of XL102 using healthy PBMCs was 4.28 μM (range 0.948–9.940 μM) which is significantly greater compared to the data obtained using primary myeloid blasts (*p* = 0.0001) (Fig. 2B-C). A higher IC_50_ value of XL102 in healthy PBMCs highlights the fact that XL102 exerts preferential therapeutic benefits in leukemic cells relative to normal cells.

Interruption of proliferation with the action of CDKs induces mitochondria-mediated apoptosis by targeting BCL2 family of proteins [7, 8]. Using, Annexin/PI staining, we found apoptosis was induced in dose-dependent manner after 24 h of XL102 treatment (Fig. 2D). Furthermore, the protein levels of MCL1, BCL-XL and XIAP have significantly reduced in dose-dependent manner. The induction of cleaved caspase3 and PARP cleavage were observed following CDK7 inhibition (Fig. 2E; Supplementary Fig. 2A). The results derived from primary myeloid blasts confirm the pro-apoptotic potential of XL102 in concordance with AML cell line data (Fig. 2F; Supplementary Fig. 2B). Taken together, these findings suggest that CDK7 inhibition by XL102 induces antiproliferative effects leading to mitochondrial mediated apoptosis in AML.

### CDK7 depletion targets cell cycle via c-Myc/p53 axis and mimics the effects of XL102

#### XL102 targets cell cycle proteins

**Fig. 3:**
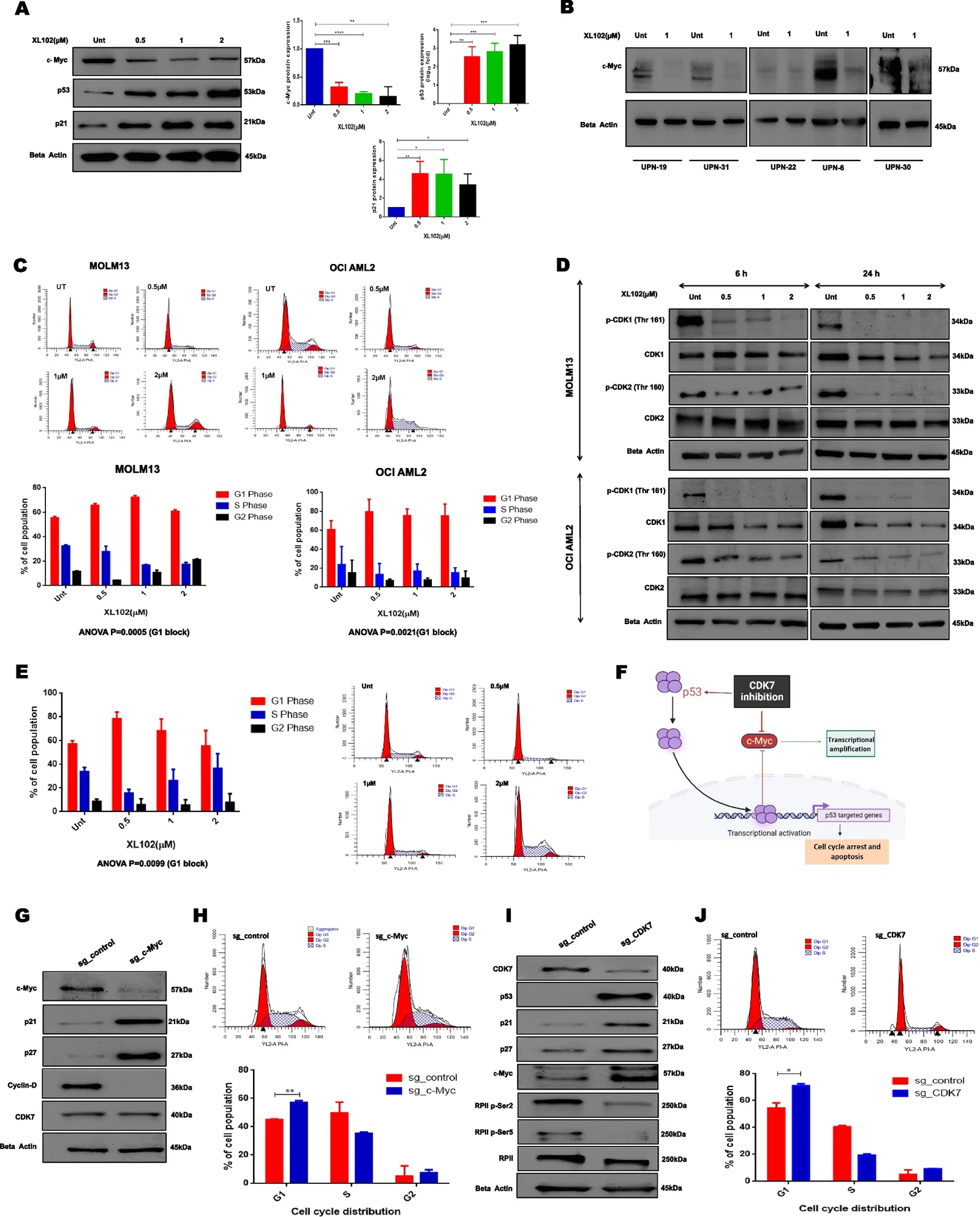
CDK7 inhibition modulates cell cycle by targeting cell cycle checkpoints. **A** Dose dependent decrease in expression of c-Myc and increase in p53 and p21 after 6 h of XL102 treatment in leukemic cells. **B** Decrease in expression of c-Myc in patient derived AML blasts after 24 h of 1 μM XL102 treatment **C** Cell-cycle analysis was performed analysis using flow cytometry on MOLM13 and OCI AML2 cells treated with XL102 for 24 h. The data showed increase in population of cells in G1 phase and significant decrease in S phase after treatment. The changes in cell-cycle distribution were significant in both cell lines (MOLM13, *P* = 0.0005; OCI AML2, *P* = 0.0021) **D** Decrease in expression p-CDK1 (Thr-161) and p-CDK2 (Thr-160) in OCI AML2 & MOLM13 cells after 6 h and 24 h of XL102 treatment. **E** Cell-cycle analysis was performed using flow cytometry on MOLM13 after serum starvation for 24 h and mimosine treatment (40 μM) followed by 24 h of XL102 treatment. **F** Schematic representation of c-Myc/p53 axis. **G** CRISPR/Cas9 genome editing tool was utilized to performed c-Myc knockout (c-Myc^KO^) using sgRNA targeting c-Myc in MOLM13 cells. The downstream targets such as p21,p27, CyclinD were analyzed along with expression of CDK7 **H** Cell cycle suppression in Myc^KO^ cells in comparison to control. **I** Genetic depletion of CDK7 using CRISPR/Cas9 and analysis of downstream targets **J** Cell cycle suppression in CDK7^KO^ cells in comparison to control

The c-Myc is frequently dysregulated in AML and plays an important role in leukemia pathogenesis [24, 25]. However, a deeper exploration of c-Myc role in modulating cell cycle in the context of AML is still unclear. c-Myc is known to exhibit significant dependence on continuous active transcription in cancer, but direct therapeutic targeting of c-Myc has proven difficult. However, reliance on c-Myc in transcription and proliferation makes AML vulnerable to CDK7 inhibition. Therefore, we investigated if c-Myc expression is vulnerable to XL102 treatment in AML cell lines and primary myeloid blasts. We observed 6 h of XL102 treatment led to the reduction in c-Myc levels (Fig. 3A). We also observed downregulation of c-Myc protein expression in primary myeloid blasts supporting the data obtained from AML cell lines. Further, the expression of p53 and its target p21 were also found to increase in dose-dependent manner after 6 h of XL102 treatment (Fig. 3A-B).

#### XL102 leads to cell cycle arrest

CDK7 is involved in the control of the G1 to S phase transition and is necessary for the progression of cells through the S phase. CDK7 inhibitors can cause G1 arrest, S phase arrest, or a combination of both, depending on the dose and duration of the treatment. Cell cycle analysis revealed that XL102 treatment for 24 h led to a significant accumulation of cells in G1 phase at low doses in MOLM13 and OCI-AML2 cells; however, at higher concentration (2 μM) we observed G2/M arrest peak in MOLM13 cells. Similarly, treatment of OCI AML2 cells at higher dose resulted in slight increase in cell number in S-phase compared to low doses of XL102 (Fig. 3C). The G1 arrest can be explained in context to XL102 mediated upregulation of p21, which is a transcriptional target of p53 (Fig. 3A), resulting in the inhibition of CDK2-cyclinE complex and progression through S phase. p21 is also known to inhibit CDK2-cyclin A and CDK1-cyclin A complexes, which are required for progression through the S phase and G2 phase, respectively, and it inhibits CDK1-cyclin B complexes, thus causing accumulation in G2/M, as observed in MOLM13 cells. These effects were associated with a reduction in phosphorylation of CDK1 (Thr-161) and CDK2 (Thr-160) after XL102 treatment, consistent with the reported cyclin-activating kinase (CAK) activity of CDK7 (Fig. 3D; Supplementary Fig. 3A). To assess the effect of CDK7 inhibition on synchronized population, we observed synchronized cells treated with XL102 were found to be again arrested in G1 phase resulting in cell cycle arrest leading to reduced cell proliferation (Fig. 3E). Furthermore, CFSE staining demonstrated a high geometric mean after XL102 treatment, reflecting the accumulation of undivided cells that retained high fluorescence intensity (Supplementary Fig. 3B).

Collectively, the data suggest XL102 represses c-Myc expression and stabilizes p53, leading to p53-mediated cell cycle arrest (Fig. 3F).

#### Genetic ablation of CDK7 and c-Myc supports downstream effects of XL102

CDK7 is a key regulator of transcription and plays a critical role in the progression of the cell cycle. It is also involved in the regulation of c-Myc expression, which is frequently up-regulated in AML and promotes cell proliferation, differentiation, and survival. Thus, we knockout c-Myc and CDK7 to study the c-Myc driven oncogenic signaling and on-target effects of XL102 by creating pharmacological mimicking. After conducting a CRISPR/Cas9 mediated knockout of c-Myc and CDK7, the cells were diluted to 0.5–1 cells per 100μL followed by plating on two distinct 96-well plates (100μL/well). However, upon visual examination of the plates under the microscope after two weeks, no colonies could be identified on plates with a dilution of less than one cell per well. Consequently, we began with 100 cells in 10 mL. Following single-cell cloning by limiting dilution, surviving clones were randomly selected from both sg_c-Myc (5 clones) and sg_CDK7 (2 clones). The protein levels of c-Myc and CDK7 of each single-cell-derived clone were analyzed by immunoblotting. In case of sg_c-Myc, we selected clone 2 (C2), which showed 75% knockout efficiency. The c-Myc^KO^ caused a significant increase in the levels of p21 and p27, while cyclin D levels were reduced. The levels of CDK7 remain unchanged in c-Myc^KO^ clones (Fig. 3G; Supplementary Fig. 3C). Further, we found c-Myc^KO^ cells were arrested in the G1 phase of cell cycle compared to the control (Fig. 3H).

In case of CDK7, most of the clones did not survive except two out of which only clone 1 (C1) showed modest expression of CDK7. The clone 1 (C1) also showed low proliferation compared to the control as assessed by CellTiter Glo assay. The reduced expansion ability of CDK7^KO^ clones can be attributed to its essential nature in regulating global transcription. However, C1 of CDK7 showed distinct phenotypic effects on downstream effector molecules compared to control. Previous reports suggested that cells with complete CDK7^KO^ are unlikely to grow and CDK7 protein is essential for cell proliferation [26, 27].

In concordance with XL102 effects on transcriptional repression and cell cycle proteins, we observed a significant reduction in the phosphorylation of Ser2 and Ser5 in the CTD domain of RPII in CDK7^KO^ cells compared to the control. However, following the genetic ablation of CDK7, we did not find a statistical difference in the c-Myc levels between control and CDK7^KO^ cells (Fig. 3I). One possible explanation for this outcome is because c-Myc is a highly complex and tightly regulated transcription factor and its regulation involves multiple other factors and other kinases [28]. The genetic depletion of CDK7 alone is not sufficient to decease expression levels of c-Myc. Our data has also shown cell cycle arrest in CDK7^KO^ cells as compared to the control. The genetic depletion of CDK7 augments p53 protein expression, leading to stabilizing its downstream targets p21 and p27 (Fig. 3I; Supplementary Fig. 3D) and lower cell proliferation in CDK7^KO^ cells compared to control (Supplementary Fig. 3E). In agreement with the effects of XL102 treatment on cell cycle, CDK7^KO^ cells were also found to be arrested in G1 phase of cell cycle (Fig. 3J).

Taken together, upregulation of p53 via XL102 mediated repression of c-Myc suggests proliferation control of AML cells as a targeted function of CDK7 inhibition.

### XL102 has shown potent anti-AML activity in vivo

#### Subcutaneous xenografts of AML

**Fig. 4:**
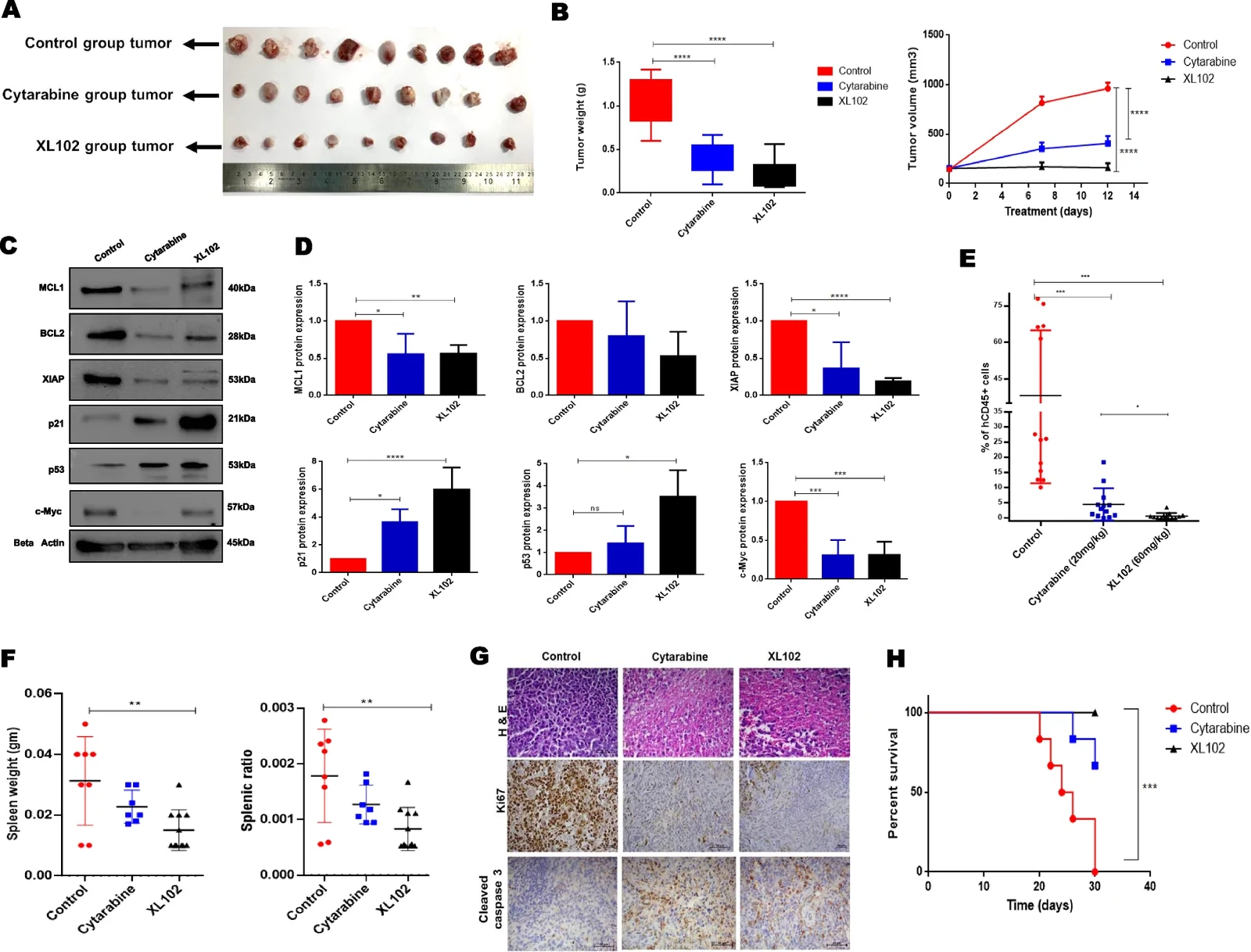
Efficacy of XL102 *in-vivo* AML model. In subcutaneous model, 2 million MOLM13 cells were injected in flank region. Ten days after establishing tumor, mice were randomized and treated with XL102 (60 mg/Kg/oral daily) and Cytarabine (20 mg/Kg/intraperitoneal daily) for 12 days. **A** Representative image of tumor from mice treated with vehicle control, Cytarabine and XL102 **B** Mean tumor weight (in grams) and mean tumor volume of mice treated with vehicle control, Cytarabine and XL102 (*n* = 9 in each group). **C**-**D** Determination of apoptosis and cell cycle markers from tumor excised from xenografts and the quantification of blots. For orthotopic model, after confirming the successful AML engraftment, mice were randomized into three groups and treated with XL102 (60 mg/Kg/oral daily) and Cytarabine (20 mg/Kg/intraperitoneal daily) for 12 days **E** Decrease in expression of hCD45 in bone marrow of treated animal groups in comparison to control. **F** Changes in spleen weight and splenic ratio. **G** H&E and immunohistochemistry staining of Ki67 and CC3 of spleen sections from vehicle or drug treated animals. Original magnification, 40X. **H** Kaplan–Meier curve showing overall survival of mice xenotransplanted with MOLM13 AML cells and treated with control, Cytarabine or XL102. Statistical significance was calculated using log-rank (Mantel–Cox) test (*P* = 0.0004; *n* = 6 per arm)

In comparison to control and Cytarabine treated AML xenografts, we observed significant tumor reduction in XL102 treated mice (Fig. 4A-B). H&E staining revealed that tumor taken from XL102 treated mice showed more apparent disintegrated cells as compared to Cytarabine and control group. (Supplementary Fig. 4A). No significant changes in the body weight of animals were observed between treated vs control groups (Supplementary Fig. 4B). The levels of anti-apoptotic proteins like MCL1, BCL2 and XIAP was reduced in tumor of animals treated with XL102 while expression of p21 and p53 was enhanced in treated group. We also found a substantial decrease in c-Myc expression in XL102-treated animals (Fig. 4C-D). These results further strengthen anti-leukemic data of XL102 in inducing apoptosis and cell cycle arrest which we obtained from AML cell lines and primary myeloid blasts.

#### Orthotopic xenografts of AML

Concerning orthotopic AML xenografts, the percentage of MOLM13 cells was quantified in murine peripheral blood using hCD45 antibody (Supplementary Fig. 4C). XL102 treated AML xenografts (*n* = 13/group) showed a remarkable reduction in hCD45 + cells (mean 0.6%; range 0.04%-3.56%) as compared to Cytarabine (mean 4.50%; range 2.10%-6.70%) and vehicle control (mean 38.2%; range 10.1%-78%) in the bone marrow (Fig. 4E). We observed significant reduction in spleen weight and splenic ratio (spleen-to-body-weight ratio) in the XL102 treated group of animals compared to control (*p* = 0.004 and 0.003, respectively (Fig. 4F). Both the treatments (Cytarabine and XL102) were well tolerated, as the changes in the percentage body weight measured at multiple time-points of treatment were not significant (Supplementary Fig. 4D). Histological analysis of tumors from XL102 treated mice revealed widespread necrotic cells, decreased cell proliferation marker Ki-67, and increased expression of the apoptotic marker cleaved caspase-3 (Fig. 4G). In another set of experiments, MOLM13 transplanted mice were divided into 3 groups and treated with vehicle control (group 1), Cytarabine (20 mg/kg) (group 2) and XL102 (60 mg/kg) (group 3) for 12 days. All the animals were then followed up for survival analyses. The median overall survival (OS) for group 1 was 25 days while OS was not reached for groups 2 and 3 animals. Of note, 4 out of 6 mice were alive in group 2 and all 6 in group 3 remained alive till 30 days after treatment (Fig. 4H).

### XL102 and Venetoclax demonstrates synergistic anti-leukemic activity in Venetoclax resistant AML cells

#### Genome wide impact of XL102 on Ven^S^ and Ven^R^ MOLM13 cells

**Fig. 5:**
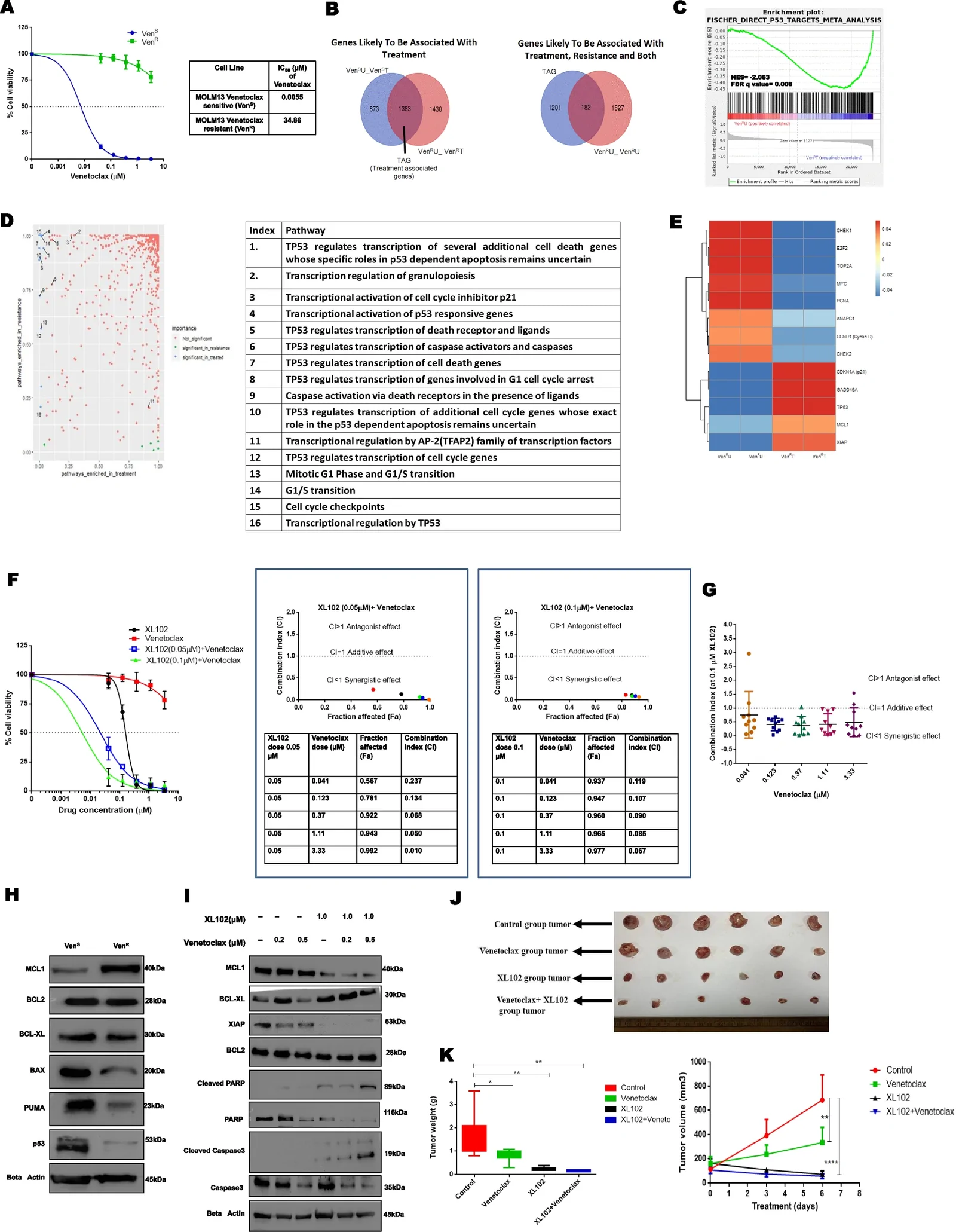
Co-operativity between XL102 and Venetoclax. **A** IC_50_ values of Venetoclax in Venetoclax sensitive (Ven^S^) and Venetoclax resistant cells (Ven^R^). **B** Venn diagram showing common genes associated with treatment and are compared with the significantly deregulated genes found between the sensitive untreated and resistant untreated. **C** GSEA plot showing the enrichment of genes related to p53 mediated signaling in Ven^R^ cells after XL102 treatment for 24 h. The Normalized Enrichment Score (NES) was found to be -2.0638618 and FDR q-value was 0.0087 **D** Principle component analysis of Ven^R^ cells after 24 h of XL102 treatment and the pathways enriched in treatment **E** Heatmap representation of deregulated genes associated exclusively with treatment in Ven^R^ **F** To investigate pharmacological interactions between XL102 and Venetoclax, incremental doses of Venetoclax were applied while the dose of XL102 was kept constant at 0.05 μM or 0.1 μM. The cell growth inhibition data indicated that combination of the drugs is highly synergistic as demonstrated by Chou-Talalay method of combination index (CI) analysis **G** AML blasts were treated with 0.1uM of XL102 along with 5 different concentrations of Venetoclax followed by determination of Combination index. **H** Basal level expression of apoptotic proteins and p53 in MOLM13 Venetoclax sensitive versus resistant cells. **I** Change in expression of antiapoptotic protein after combinatorial treatment with XL102 and venetolcax in Ven^R^ cells **J** Representative image of tumors from animals treated with combination of Venetoclax and XL102. **K** Venetoclax and XL102 combination treatment resulted in reduction of mean tumor weight (in grams) and mean tumor volume of AML xenografts as compared to control, Venetoclax alone or XL102 alone

To identify the transcriptional targets of XL102 and modulation of major cancer pathways following XL102 treatment, we used paired Ven^S^ and Ven^R^ MOLM13 cells for RNA sequencing**.** The RNA sequencing data have been submitted to Gene Expression Omnibus (GEO accession number GSE231745). The IC_50_ values of Venetoclax in Ven^S^ and Ven^R^ cells were 0.005 μM and 34.86 μM, respectively (Fig. 5A). Given that Ven^R^ cells will have differential gene expression profiles compared to Ven^S^ even without XL102 treatment, we adopted a two-stage analysis to identify genes exclusively associated with XL102 treatment in MOLM13 Ven^R^ cells. Stage 1 gene expression analyses were done between Ven^S^ and Ven^R^ cells with or without XL102 to identify common treatment-associated genes (TAG, *n* = 1383). During stage 2 analyses, TAG were further scanned in the gene expression data sets derived from untreated Ven^S^ and Ven^R^ cells. These analyses resulted in the identification of genes exclusively associated with XL102 treatment (*n* = 1201), Venetoclax resistance (*n* = 1827), and both treatment as well as resistance (*n* = 182) (Fig. 5B). The list of differentially regulated genes is provided in Supplementary Table 2 and additional details are given in Supplementary Fig. 5A and B. The detailed protocol of quality control of raw Fastq files, alignment and quantification of the reads, and pathway analysis has been provided in supplementary data.

Gene set enrichment analysis (GSEA) of XL102 treated cells revealed the enrichment of genes that are known to be p53 target (Fig. 5C). To establish the correlation between p53 mediated signaling and CDK7 inhibition, the RNA sequencing data of XL102 treated versus untreated cells were re-analyzed by principal component analysis (PCA). The results indicated the expression of gene transcripts involved in cell cycle and apoptotic pathways (p21, c-Myc, TP53, MCL1, XIAP) were significantly modulated after XL102 treatment (Fig. 5D-E). Heatmap of Ven^S^ cells treated with XL102 is provided in Supplementary Fig. 5C. The transcripts levels of the key genes involved in these pathways such as p21, BTG2, CHEK1, GADD45, PCNA, INKA2, TOP2A, E2F1, MCL1, BCL-XL and c-Myc were also validated using real-time quantitative PCR (RQ-PCR) at 6 h and 24 h of XL102 treatment (Supplementary Fig. 5D).

#### XL102 synergizes with Venetoclax and can overcome Venetoclax resistance

To determine if the combination of XL102 and Venetoclax can sensitize the Ven^R^ cells to undergo apoptosis, we co-treated these cells with XL102 and Venetoclax. The combination treatment potently reduces cell proliferation as compared to single agent. Additionally, XL102 and Venetoclax showed remarkable synergy as CI values were found to fall below 1 across all combinations (Fig. 5F). Further, XL102 and Venetoclax combination decreases cell proliferation and enhances apoptosis in AML cell lines (Supplementary Fig. 5E-F). Next, we analyzed ex vivo anti-proliferation data of primary blasts collected from 10 AML patients at baseline. Individual IC_50_ of each patient along with the combination index values has been provided in Supplementary Table 3. As observed in AML cell lines, we found synergistic effects in primary myeloid blasts (Fig. 5G). Taking the lead from RNA sequencing data of Ven^S^ and Ven^R^ cells, we next examined the expression levels of BCL2 family proteins and p53. Consistent with previous reports [29, 30], we also found that MCL1 is significantly upregulated whereas BAX, PUMA and p53 are downregulated in Venetoclax resistant cells as compared to the sensitive counterpart (Fig. 5H; Supplementary Fig. 5G). We next treated Ven^R^ cells with Venetoclax alone or in combination with XL102. The expression levels of MCL1 and XIAP were significantly reduced while protein levels of BCL-XL were mildly affected on combined treatment. The enhanced levels of cleaved PARP and cleaved Caspase 3 were observed in combination of XL102 and Venetoclax (Fig. 5I; Supplementary Fig. 5H).

Further, to test the efficacy of XL102 and Venetoclax in vivo, we injected 2 million Venetoclax-resistant cells subcutaneously NOD/SCID mice. After 12 days of tumor formation, mice were randomized into four arms and dosed orally daily with control (0.1%DMSO), Venetoclax (100 mg/kg), XL102 (60 mg/kg) or Venetoclax in combination with XL102 daily for six days. The animals were sacrificed and tumor was harvested for lysate preparation. As shown in Fig. 5J-K; Supplementary Fig. 5I-J, inhibition of tumor growth and tumor regression was significantly greater in AML xenografts treated in combination with Venetoclax and XL102 as compared to control or Venetoclax alone or XL102 alone. Taken together, these findings confirmed that combination of CDK7 and BCL2 via XL102 reduces proliferation, amplifies apoptosis and found to be highly synergistic in AML cell lines, primary myeloid blasts and AML xenografts.

## Discussion

Some of the current challenges in AML drug development are high reliance on conventional 3 + 7 treatment as historical precedent in achieving induction remission. The suboptimal outcomes of 3 + 7 conventional therapy in AML over 40 years have finally struck a chord resulting in the fast-track approval of 10 new agents in the past five years [31, 32]. Although AML blasts display tremendous genetic heterogeneity (Table 1), these blasts remain exquisitely addicted to oncogenes responsible for high proliferation and transcriptional rates, such as c-Myc and MCL1. CDK7 is an attractive target for inhibitors that kill tumor cells by exploiting high cell proliferation and tumor-specific transcriptional dependencies of oncogenes [24, 33].

In this study, the functional and mechanistic data derived from XL102, an orally bioavailable covalent inhibitor of CDK7, provide substantial preclinical precedence for treating AML by simultaneously inhibiting cell cycle progression and transcriptional activity of oncogenes leading to apoptosis. CDK7 phosphorylation of RNA polymerase II at Serine 2,5 and 7 within its C-terminal domain is required for the recruitment of other factors, including the capping enzyme and spliceosome components, to the nascent transcript. These factors are essential for the processing and stabilization of the RNA transcripts. However, CDK7 inhibitors can cause a decrease in basal levels of RNA polymerase II by inhibiting its phosphorylation and leading to its destabilization even in the absence of transcriptional stimulation (Fig. 1C).

CDK7 inhibition by XL102 affects the expression levels of phospho- and total CDK1 and CDK2. Following treatment with XL102, a decrease in the levels of CDK1 (Thr161), CDK2 (Thr160), as well as total CDK1 and CDK2, was observed in MOLM13 and OCIAML2 cells. Notably, we observed a more pronounced reduction in the protein levels of CDK1 compared to CDK2 in both cell lines. Previous studies [34, 35] involving leukemia and breast cancer models have revealed that CDK1 relies more on CDK7-mediated activation activity and stability, while other CDK-activating kinases can also regulate CDK2, in addition to CDK7. In the study by Patel et al., [35] utilizing ICEC0942 (a CDK7 inhibitor), a more prominent reduction in CDK1 levels, as compared to CDK2, was observed in tumor responses to CDK7 inhibition. Likewise, Park et al., [34] demonstrated that CDK7 inhibition led to reduced levels of CDK7-directed activating phosphorylation of CDK1 (Thr161) and CDK2 (Thr160), along with a decrease in the resulting CDK1/2, in leukemic cells. Besides, the ability of XL102 to modulate MCL1 levels in primary AML blasts, Venetoclax resistant cells, and xenograft models and observed synergistic anti-leukemic activities of combined XL102 and Venetoclax treatment may have therapeutic benefits in a genetically heterogeneous disease like AML. At the therapeutic dose of 60 mg/kg of XL102 in AML xenografts, we did not observe any significant adverse side effects even at daily oral treatment for 12 days. We observed a significant reduction in Ki67 expression and upregulation of cleaved Caspase 3 in AML xenografts treated with XL102 as compared to the control group. These results were in agreement with the anti-proliferative and pro-apoptotic activities of XL102 observed in vitro.

THZ1, the prototype of CDK7 inhibitor, has shown excellent anticancer effects but lacks oral bioavailability. Consequently, YKL-5–124 was developed, similar to THZ1, covalently links to Cys312 of CDK7 but does not affect the activities of CDK12 and 13 [36]. Further, to enhance the potency, stability, and selectivity, the THZ1-derived CDK7 inhibitor, SY-1365 followed by SY-5609 were developed by Syros Pharmaceuticals [8]. Four CDK7 inhibitors, including XL102, have entered clinical trials to treat various metastatic solid tumors (NCT03134638, NCT04247126, NCT03770494, NCT04726332).

Using an AML cell line and two distinct genetically engineered mouse models of AML, Minzel et al. [37] demonstrated that CDK7 inhibition led to p53 activation followed by induction of apoptosis. THZ1 induces cell apoptosis in c-Myc overexpressed cells in acute lymphoblastic leukemia via p53 upregulation [10]. Similarly, XL102 treatment in AML cells leads to p53 stabilization resulting in cell cycle arrest at G1/S phase. Furthermore, pharmacological inhibition by XL102 was mimicked by genetic ablation of CDK7 that displayed increased levels of p21 and activation of p53 in CDK7^KO^ AML cells, suggesting that p53 activity is controlled by CDK7 [38]. These results provide an additional mechanism of p53 regulation via CDK7 targeting in AML to the existing data on the targeting of p53 using MDM2 inhibitors to eliminate AML blasts [39]. p53 along with c-Myc controls cell survival and proliferation to maintain cellular homeostasis by interacting in a negative feedback manner. In line with previously published data [40–42] we have also shown that CDK7 inhibition by XL102 decreases c-Myc levels in a dose-dependent manner. CDK7 is dispensable for phosphorylation of the conserved Ser5 residue located in the CTD of RNA polymerase II and for regulating global transcription. It has been reported that other CDKs, such as CDK9, have been shown to phosphorylate Ser5 in the CTD of RNA polymerase II which may compensate for the loss of CDK7 [26, 43]. As a result, the genetic ablation of CDK7 may not significantly affect c-Myc expression (Fig. 3H). However, more research is needed to fully understand the impact of CDK7 genetic ablation on c-Myc expression and function. On the other hand, CDK7 inhibitors are drugs that specifically inhibit the kinase activity of CDK7. This would result in the decreased phosphorylation of RNA polymerase Il and, consequently, a decrease in the transcription of genes, including c-Myc. Therefore, the use of CDK7 inhibitor can downregulate c-Myc levels, as observed from our in vitro, ex vivo and in vivo data. The downstream targets such as p21 and p27, known to induce proliferation arrest in G1/S, were found to be upregulated in c-Myc^KO^ cells.

Several MCL1-specific inhibitors have been developed to rescue Venetoclax resistance; however, simultaneous targeting of MCL1 and BCL-2 remains a concern in a normal cell [29, 44–46]. Therefore, XL102 has additional value in AML as it reduces MCL1 levels in primary myeloid blasts, Venetoclax sensitive, and resistant MOML13 cells. Interestingly, we noticed that combination treatment of Venetoclax resistant MOLM13 cells decreases the levels of anti-apoptotic proteins and upregulates the expression of proapoptotic proteins. Furthermore, RNA sequencing data of XL102 treated MOLM13 cells have shown MCL1 and BCL-XL as transcriptional targets of CDK7, which was further confirmed by real-time PCR. Taken together, this data suggests that XL102 has the ability to overcome Venetoclax resistance.

In conclusion, our study provides extensive preclinical evidence that XL102 induces p53-mediated elimination of leukemic cells via CDK7/c-Myc/p53 axis. Combined treatment with XL102 and Venetoclax led to synergistic anti-leukemic effects and modulation of the BCL2 family of proteins in Venetoclax-resistant cells.

## Supplementary Information

**Additional file 1:** **Supplementary Table 1.**

**Additional file 2:** **Supplementary Table 2.**

**Additional file 3:** **Supplementary Table 3.**

**Additional file 4:** **Supplementary Figure 1.** Change in protein expression of CDK7 and RPII after XL102 treatment- Quantification of Western blot data at 6hrs and 24hrs of drug treatment. Results are the mean ± SD of three independent experiments. *P* values < 0.05 were considered significant, where *p* value < 0.05 (^∗^), *p* value < 0.01(^∗∗^), *p* value < 0.001 (^∗∗∗^), *p* value < 0.0001 (^∗∗∗∗^). **Supplementary Figure 2.** XL102 treatment leads to apoptosis: (A) The levels of pro-apoptotic and anti-apoptotic protein was quantified after 24hrs of XL102 treatment in MOLM13 as well as OCI AML2 cell lines. (B) The levels of MCL1, XIAP and BCL-XL in patient derived AML blast were quantified after 24hrs of XL102 treatment. **Supplementary Figure 3.** CDK7 depletion leads to modulation of cell cycle: (A) The levels of phospho CDK1and CDK2 decreases in dose dependent manner after 6hrs and 24hrs of treatment. (B) CFSE staining of leukemic cells after 24hrs of XL102 treatment. Geometric mean or mean fluorescence intensity of dye within the cells increases on drug treatment indicating proliferation arrest. (C) Protein quantification of c-Myc, p21,p27, CyclinD and CDK7 in c-Myc^KO^ cells versus control. (D) Protein quantification of CDK7, p53, p21, p27 and Ser2/5/RPII in CDK7^KO^ versus control (E) Though the proliferation rate in CDK7^KO^ cells in comparison to MOLM13 cells was slightly decreased, there was no statistical difference (*p*=0.48, *n*=3). **Supplementary Figure 4.** Effect of XL102 treatment on AML animal models (A) Representative image of H&E-stained tumor sample from each group after 12 days of drug treatment (B) Change in body weight of animals in different groups. (C) Representative image of confirmation of engraftment using dot plot of mCD45 and hCD45 in peripheral blood and bone marrow of animals. (D) Percentage changes in body weight of animals in different groups. **Supplementary figure 5.** Combination of XL102 with Venetoclax shows synergy in AML cells. (A) Pearson’s correlation matrix based on the gene expression showing the exclusiveness of all four conditions (Parental untreated, parental treated, resistant untreated and resistant treated). There is a higher correlation found between the parental and resistant samples respectively. (B) MA plot representing differential analysis of the three comparisons (Parental untreated vs resistant untreated, parental untreated vs parental treated and resistant untreated vs resistant treated) and highlighting the significantly up or down-regulated genes in each of them. (C) GSEA analysis of Ven^S^ cells showing differentially regulated genes. (D) Q-PCR analysis of modulated genes after XL102 treatment for 24hrs. (E) The combinatorial treatment of XL102 with Venetoclax shows synergy in OCI AML2 cells as the combination index values were less than 1 in all combinations. (F) Enhanced apoptosis was observed after a combination of XL102 and Venetoclax using Annexin/Pi staining in AML cells. (G) Western blot quantification of basal level expression of apoptotic proteins and p53 in MOLM13 Venetoclax sensitive versus resistant cells (H) Quantification of western blots of apoptosis protein after combination of XL102 and Venetoclax in MOLM13 Venetoclax resistant cells. (I-J) Determination of apoptosis markers from tumor excised from xenografts and the quantification of blots.

## Data Availability

All the original data including immunoblots (triplicates) are available with corresponding author and can be provided on request.
