# Correction: Novel covalent CDK7 inhibitor potently induces apoptosis in acute myeloid leukemia and synergizes with Venetoclax

**DOI:** 10.1186/s13046-026-03824-1

**Published:** 2026-09-18

**Authors:** Tarang Gaur, Ramulu Poddutoori, Leena Khare, Bhausaheb Bagal, Sonal Rashmi, Nikhil Patkar, Prashant Tembhare, Subramanian PG, Dhanlaxmi Shetty, Amit Dutt, Qi Zhang, Marina Konopleva, Uwe Platzbeckar, Sueep Gupta, Susanta Samajdar, Murali Ramchandra, Navin Khattry, Syed K. Hasan

**Affiliations:** 1 Hasan Lab, Advanced Centre for Treatment, Research and Education in Cancer (ACTREC), Tata Memorial Centre, Navi, Mumbai, 410210 India; 2 Homi Bhabha National Institute (HBNI),, Anushaktinagar, Mumbai, 400094 India; 3 Aurigene Oncology Limited, Electronic City, Hosur Road, Bangalore, 560100 India; 4 Department of Medical Oncology, Tata Memorial Centre, Tata Memorial Hospital, Mumbai, 400014 India; 5 Dutt Lab, Advanced Centre for Treatment, Research and Education in Cancer (ACTREC), Tata Memorial Centre, Navi, Mumbai, 410210 India; 6 Present Address: CNAG-CRG, Centre for Genomic Regulation (CRG), Barcelona Institute of Science and Technology (BIST), Barcelona, Spain; 7 Hematopathology Laboratory, Advanced Centre for Treatment, Research and Education in Cancer (ACTREC), Tata Memorial Centre, Navi, Mumbai, 410210 India; 8 Department of Cytogenetics, Advanced Centre for Treatment, Research and Education in Cancer (ACTREC), Tata Memorial Centre, Navi, Mumbai, 410210 India; 9 Department of Leukemia, The University of Texas MD Anderson Cancer Center, Houston, TX 77030 USA; 10 Albert Einstein College of Medicine, Bronx, NY 10461 USA; 11 Medical Clinic and Policlinic I, Hematology and Cellular Therapy, University Hospital Leipzig, Johannisallee 32, Leipzig, 04103 Germany

**Correction: J Exp Clin Cancer Res 42, 186 (2023)**

**https://doi.org/10.1186/s13046-023-02750-w**

Following publication of the article [1], the authors identified an error in the published version of Fig. 2F, XIAP Western blot panel should be labeled UPN-30, UPN-22, UPN-6, and UPN-26 instead of UPN-6, UPN-22, UPN-30, and UPN-26.

Incorrect Fig. 2F

**Fig. 2:**
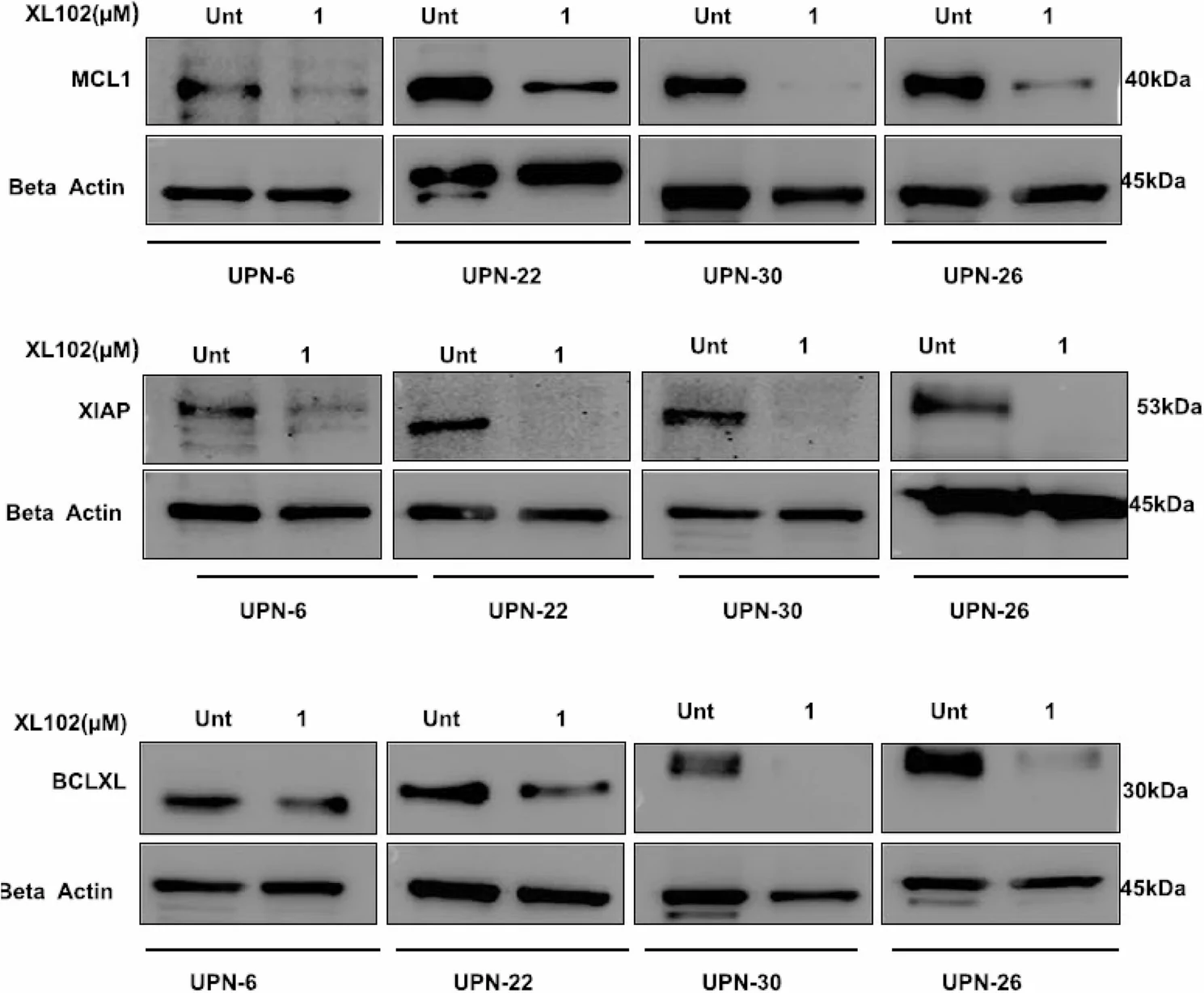
Anti-leukemic activity of XL102 in AML cell lines, primary myeloid blasts and healthy individuals. **A** The antiproliferative effect of XL102 using different AML cells was determined using CTG assay after 72 h of drug treatment. Values were calculated using GraphPad Prism by log-transforming the data and fitting it to a nonlinear regression. **B** Antiproliferative analysis of XL102 in patient derived myeloid blasts of de novo (*n* = 42), relapsed/refractory (*n* = 12) and mononuclear cells derived from healthy individuals (*n* = 15). **C** Comparative analyses of XL102 cytotoxicity in AML blast and healthy PBMC. **D** Cell apoptosis was measured using a flow cytometry analysis of Annexin/PI staining after 24 h of XL102 treatment at the indicated concentrations in MOLM13 and OCI AML2 cells. **E** Change in expression of antiapoptotic protein after 24 h of drug treatment in AML cells. Induction of cleaved PARP and cleaved Caspase-3 in dose dependent manner. **F** Change in expression of antiapoptotic protein in patient derived blast after 24 h of drug treatment. All the western blots are performed in triplicates

Correct Fig. 2F

**Fig. 2:**
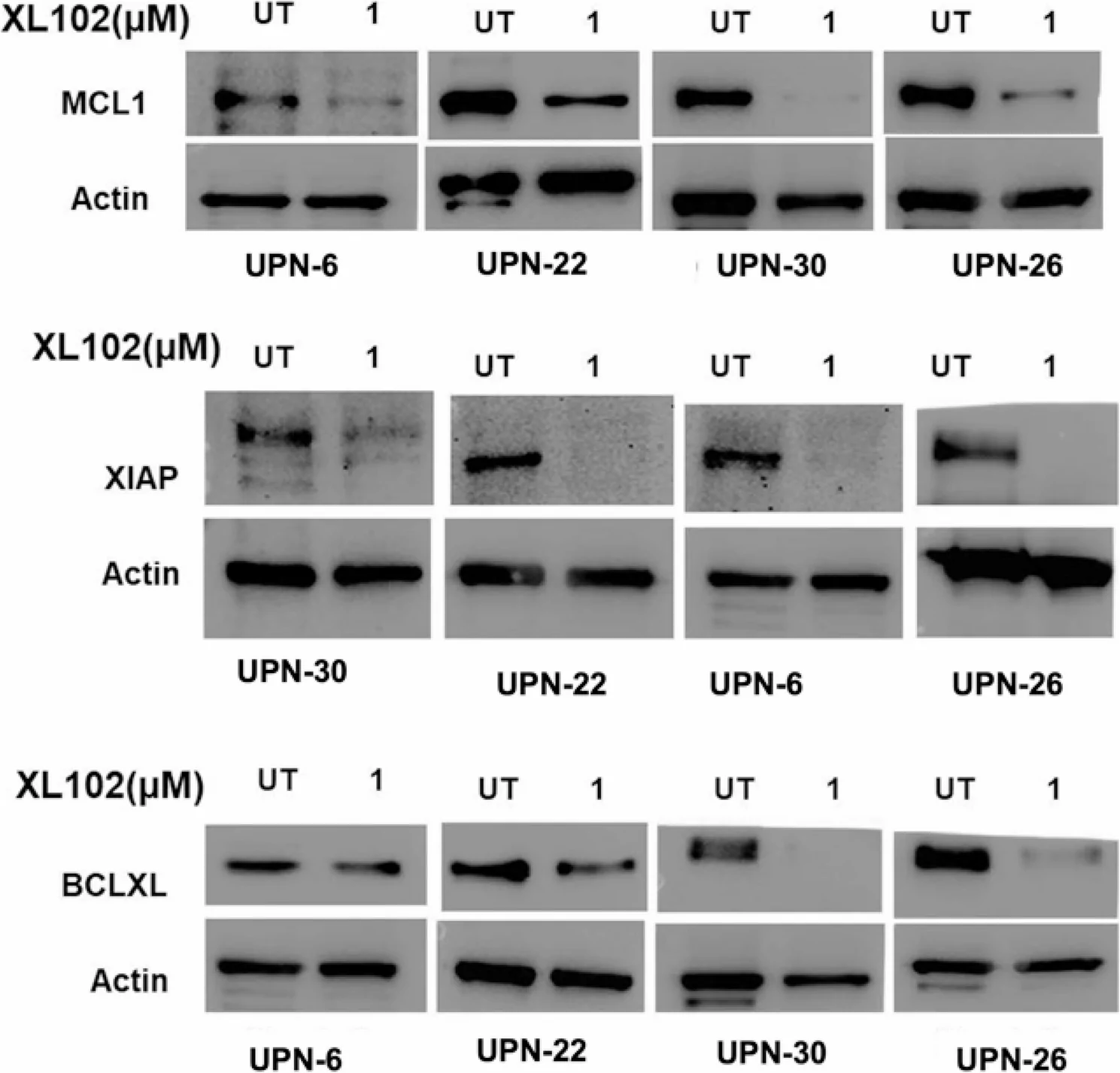
Anti-leukemic activity of XL102 in AML cell lines, primary myeloid blasts and healthy individuals. **A** The antiproliferative effect of XL102 using different AML cells was determined using CTG assay after 72 h of drug treatment. Values were calculated using GraphPad Prism by log-transforming the data and fitting it to a nonlinear regression. **B** Antiproliferative analysis of XL102 in patient derived myeloid blasts of de novo (*n* = 42), relapsed/refractory (*n* = 12) and mononuclear cells derived from healthy individuals (*n* = 15). **C** Comparative analyses of XL102 cytotoxicity in AML blast and healthy PBMC. **D** Cell apoptosis was measured using a flow cytometry analysis of Annexin/PI staining after 24 h of XL102 treatment at the indicated concentrations in MOLM13 and OCI AML2 cells. **E** Change in expression of antiapoptotic protein after 24 h of drug treatment in AML cells. Induction of cleaved PARP and cleaved Caspase-3 in dose dependent manner. **F** Change in expression of antiapoptotic protein in patient derived blast after 24 h of drug treatment. All the western blots are performed in triplicates

This correction does not affect the results, interpretation, or conclusions of the article. The original article [1] has been corrected.
