# Supplementary material for: Novel covalent CDK7 inhibitor potently induces apoptosis in acute myeloid leukemia and synergizes with Venetoclax
Source: J Exp Clin Cancer Res. 2023 Jul 29;42:186. doi: 10.1186/s13046-023-02750-w (PMC10386772; doi:10.1186/s13046-023-02750-w)
Supplement: Supplementary file 4 — Additional file 4: Supplementary Figure 1. Change in protein expression of CDK7 and RPII after XL102 treatment- Quantification of Western blot data at 6hrs and 24hrs of drug treatment. Results are the mean ± SD of three independent experiments. P values < 0.05 were considered significant, where p value < 0.05 (∗), p value < 0.01(∗∗), p value < 0.001 (∗∗∗), p value < 0.0001 (∗∗∗∗). Supplementary Figure 2. XL102 treatment leads to apoptosis: (A) The levels of pro-apoptotic and anti-apoptotic protein was quantified after 24hrs of XL102 treatment in MOLM13 as well as OCI AML2 cell lines. (B) The levels of MCL1, XIAP and BCL-XL in patient derived AML blast were quantified after 24hrs of XL102 treatment. Supplementary Figure 3. CDK7 depletion leads to modulation of cell cycle: (A) The levels of phospho CDK1and CDK2 decreases in dose dependent manner after 6hrs and 24hrs of treatment. (B) CFSE staining of leukemic cells after 24hrs of XL102 treatment. Geometric mean or mean fluorescence intensity of dye within the cells increases on drug treatment indicating proliferation arrest. (C) Protein quantification of c-Myc, p21,p27, CyclinD and CDK7 in c-MycKO cells versus control. (D) Protein quantification of CDK7, p53, p21, p27 and Ser2/5/RPII in CDK7KO versus control (E) Though the proliferation rate in CDK7KO cells in comparison to MOLM13 cells was slightly decreased, there was no statistical difference (p=0.48, n=3). Supplementary Figure 4. Effect of XL102 treatment on AML animal models (A) Representative image of H&E-stained tumor sample from each group after 12 days of drug treatment (B) Change in body weight of animals in different groups. (C) Representative image of confirmation of engraftment using dot plot of mCD45 and hCD45 in peripheral blood and bone marrow of animals. (D) Percentage changes in body weight of animals in different groups. Supplementary figure 5. Combination of XL102 with Venetoclax shows synergy in AML cells. (A) Pearson’s correlation matrix based on [file 13046_2023_2750_MOESM4_ESM.docx]

**Supplementary data**

**RNA sequencing:**

Raw reads were available as fastq files. The quality of the Illumina sequenced paired-end fastq files were assessed using Fastqc (v0.11.4). Furthermore, Trimmomatic (v0.32) was used to remove adapters and improve average read phred quality. It is achieved using 3 parameters of the software: firstly, adapter removal via ILLUMINACLIP and TruSeq3-PE-2 as illumina adapter reference, secondly HEADCROP of bad quality 10bp of each of the read and lastly sliding window with average phred quality 15 in each 10bp window. The mean length, GC content and number of reads were found to be 151, 52.5% and 87203261 respectively in the paired-end fastq file. Post QC there is an average loss of 42.38% reads. Average read length is around 100 and 50229865 total reads in each of the paired end fastq file.


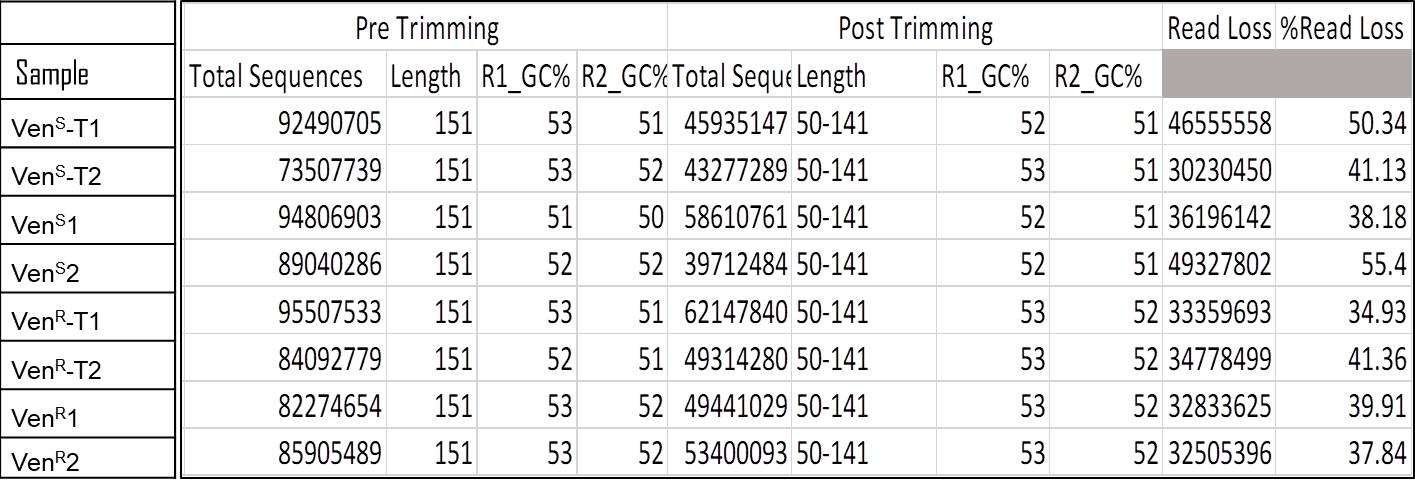


**Alignment of the reads**

The RNAseq reads were aligned to the GRCh38 human reference using Hisat(v 2.1.0). The output sequence alignment map file (sam) was converted to the binary format (bam) file using samtools (v1.9) to reduce space and increase the speed to read the same efficiently. The overall mapping quality and biases in sequencing and/or mapping of the Bam files were assessed using Qualimap (v2.2.1) according to the GRch38 annotated feature file. There were no bias found and overall mean coverage and mapping percentage across the feature genomic region is 78.46% and 508.82 X respectively.

| Sample ID | Number of mapped reads (GLOBAL) | Number of mapped reads (inside region/on-target) | Mapping percentage (inside region) | Coverage (inside region) |
| --- | --- | --- | --- | --- |
| Ven^S^-T1 | 10,50,23,488 | 9,38,73,059 | 81.74% | 614.15 X |
| Ven^S^-T2 | 9,62,90,026 | 8,67,03,229 | 82.97% | 381.55 X |
| Ven^S^1 | 13,46,75,171 | 11,88,49,358 | 79.92% | 699.26 X |
| Ven^S^2 | 9,24,23,783 | 8,04,35,657 | 78.88% | 340.76 X |
| Ven^R^-T1 | 14,95,34,924 | 12,40,71,046 | 75.71% | 485.52 X |
| Ven^R^-T2 | 12,18,21,049 | 10,36,09,249 | 78.46% | 557.05 X |
| Ven^R^1 | 11,59,20,864 | 9,74,78,738 | 74.36% | 514.70 X |
| Ven^R^2 | 12,32,77,648 | 10,53,66,805 | 75.62% | 477.58 X |

**Quantification of reads**

FeatureCounts (Subread v1.6.2) was used to quantify the mapped reads for genomic regions present in the GRch38 annotated file including chromosomal coordinates of features. The downstream analysis of the quantified read matrix is done using DeSeq.

**Differential analysis**

The differential analysis was done between parental untreated (Ven^S^) and parental treated (Ven^S^-T); resistant untreated (Ven^R^) and resistant treated (Ven^R^-T); and parental untreated (Ven^S^) and resistant untreated (Ven^R^) with parental untreated (Ven^S^), resistant untreated (Ven^R^) and parental untreated (Ven^S^) respectively being control. For each of the 3 comparisons, DESeq Data Set object is created using count matrix and metadata containing sample and condition which is used as design formula. Genes with total read count across samples less than 10 are eliminated. DeSeq is used on the objects to find differential genes, which are further filtered based on adjusted pvalue (< 0.05) and log2FC (between -1 and 1).

There are 2256, 2813 and 2009 significant genes found in differential between parental untreated (Ven^S^) and parental treated (Ven^S^-T); resistant untreated (Ven^R^) and resistant treated (Ven^R^-T); and parental untreated (Ven^S^) and resistant untreated (Ven^R^) respectively. There are 1201, 1827 and 182 significantly differentiated genes associated with treatment, resistance and both treatment and resistance.

**Supplementary Figures**


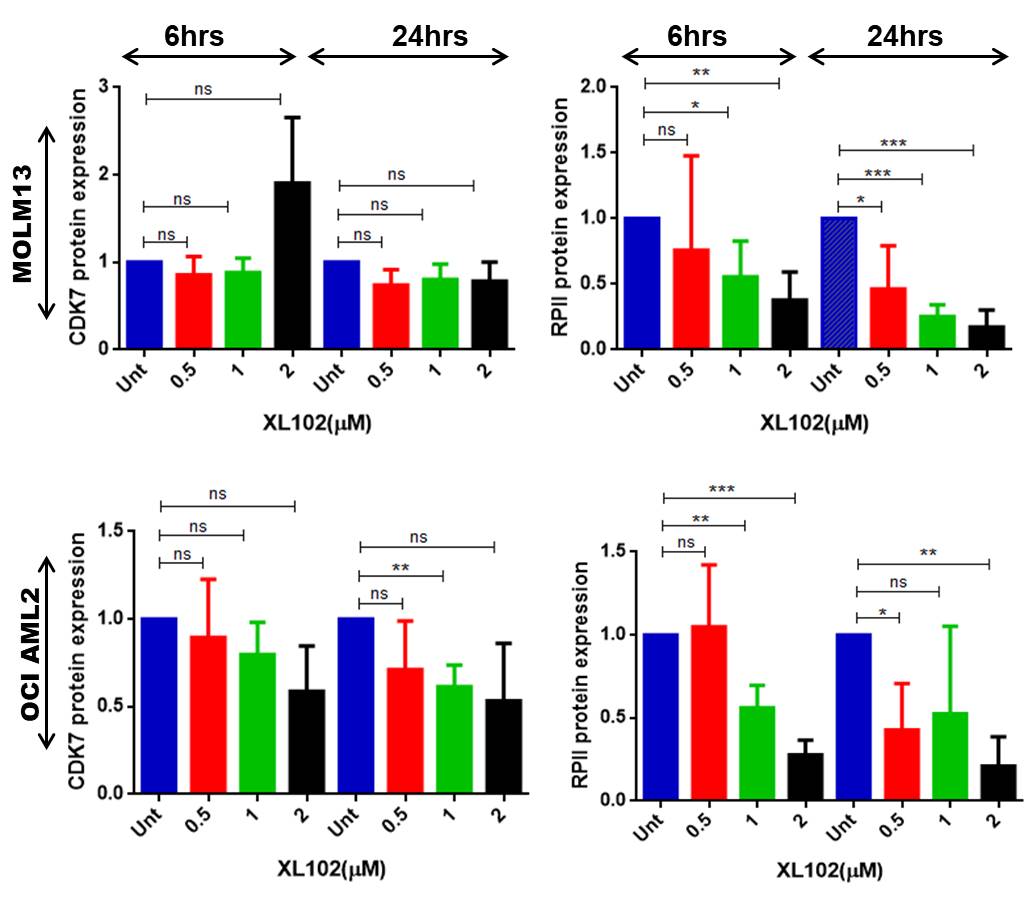


**Supplementary Figure 1: Change in protein expression of CDK7 and RPII after XL102 treatment**- Quantification of Western blot data at 6hrs and 24hrs of drug treatment. Results are the mean ± SD of three independent experiments. P values < 0.05 were considered significant, where p value < 0.05 (^∗^), p value < 0.01(^∗∗^), p value < 0.001 (^∗∗∗^), p value < 0.0001 (^∗∗∗∗^)

**
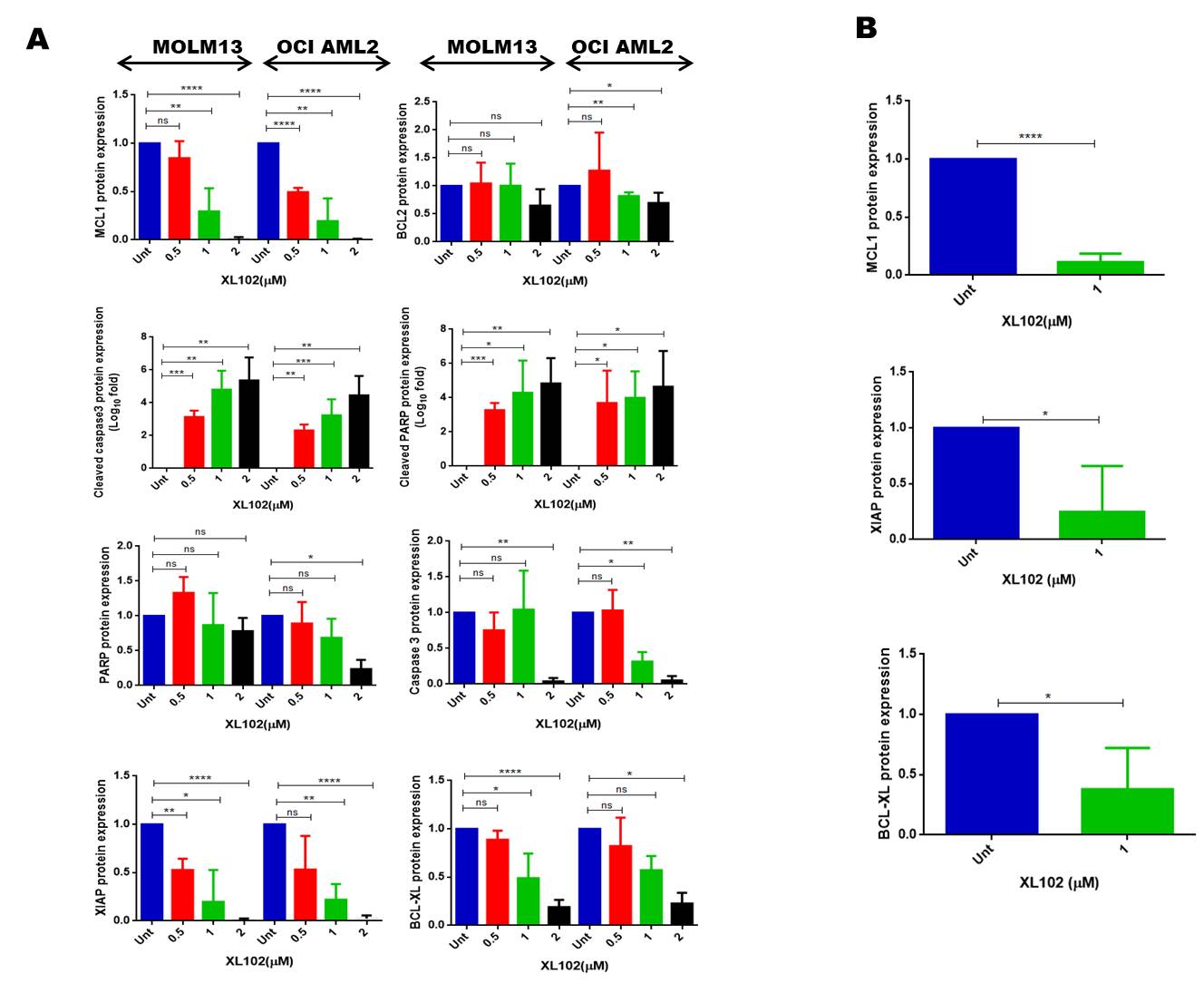
**

**Supplementary Figure 2: XL102 treatment leads to apoptosis**: (A) The levels of pro-apoptotic and anti-apoptotic protein was quantified after 24hrs of XL102 treatment in MOLM13 as well as OCI AML2 cell lines. (B) The levels of MCL1, XIAP and BCL-XL in patient derived AML blast were quantified after 24hrs of XL102 treatment.


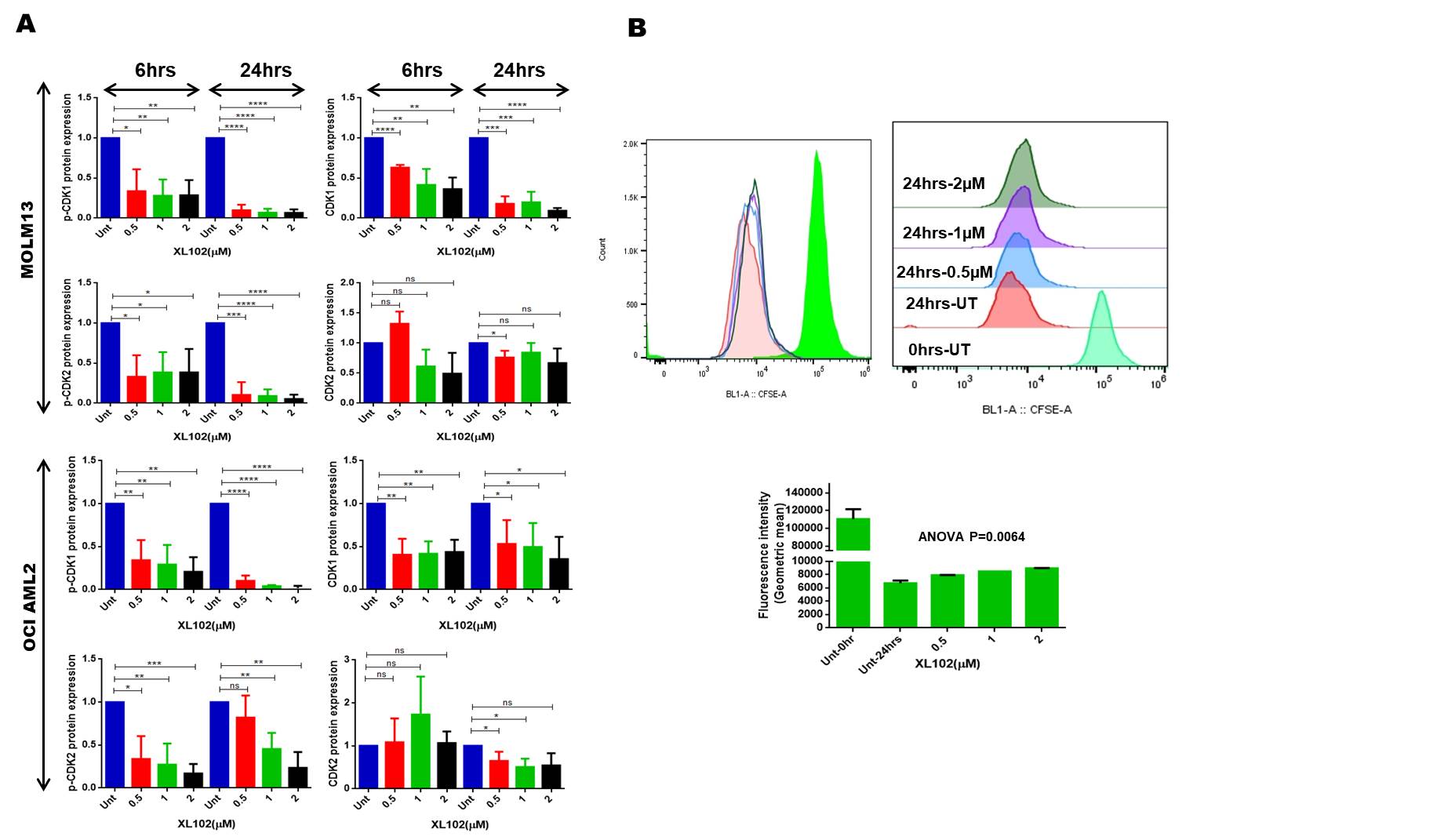


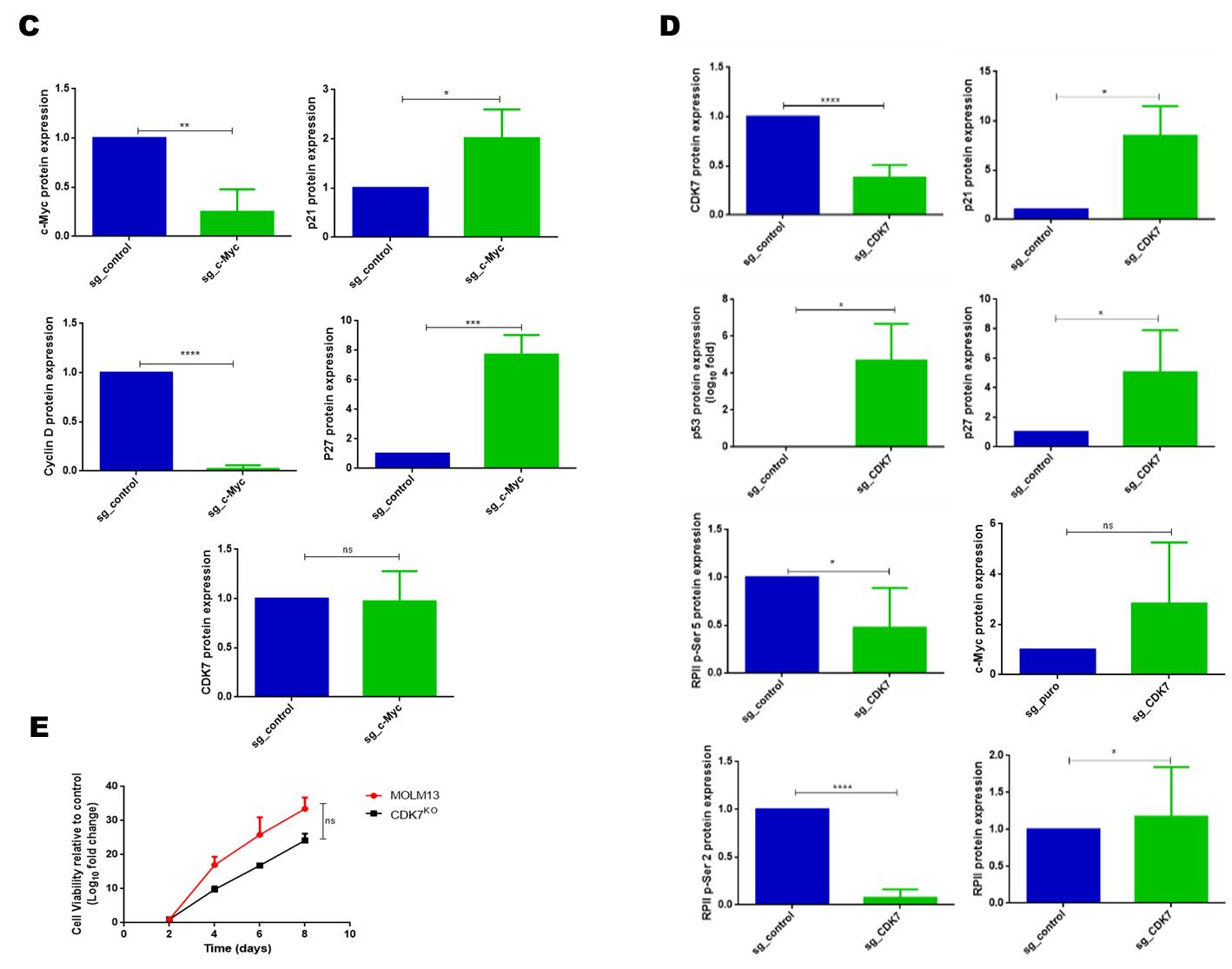


**Supplementary Figure 3: CDK7 depletion leads to modulation of cell cycle: (A)** The levels of phospho CDK1and CDK2 decreases in dose dependent manner after 6hrs and 24hrs of treatment. (B) CFSE staining of leukemic cells after 24hrs of XL102 treatment. Geometric mean or mean fluorescence intensity of dye within the cells increases on drug treatment indicating proliferation arrest. (C) Protein quantification of c-Myc, p21,p27, CyclinD and CDK7 in c-Myc^KO^ cells versus control. (D)Protein quantification of CDK7, p53, p21, p27 and Ser2/5/RPII in CDK7^KO^ versus control (E) Though the proliferation rate in CDK7^KO^ cells in comparison to MOLM13 cells was slightly decreased, there was no statistical difference (p=0.48, n=3)


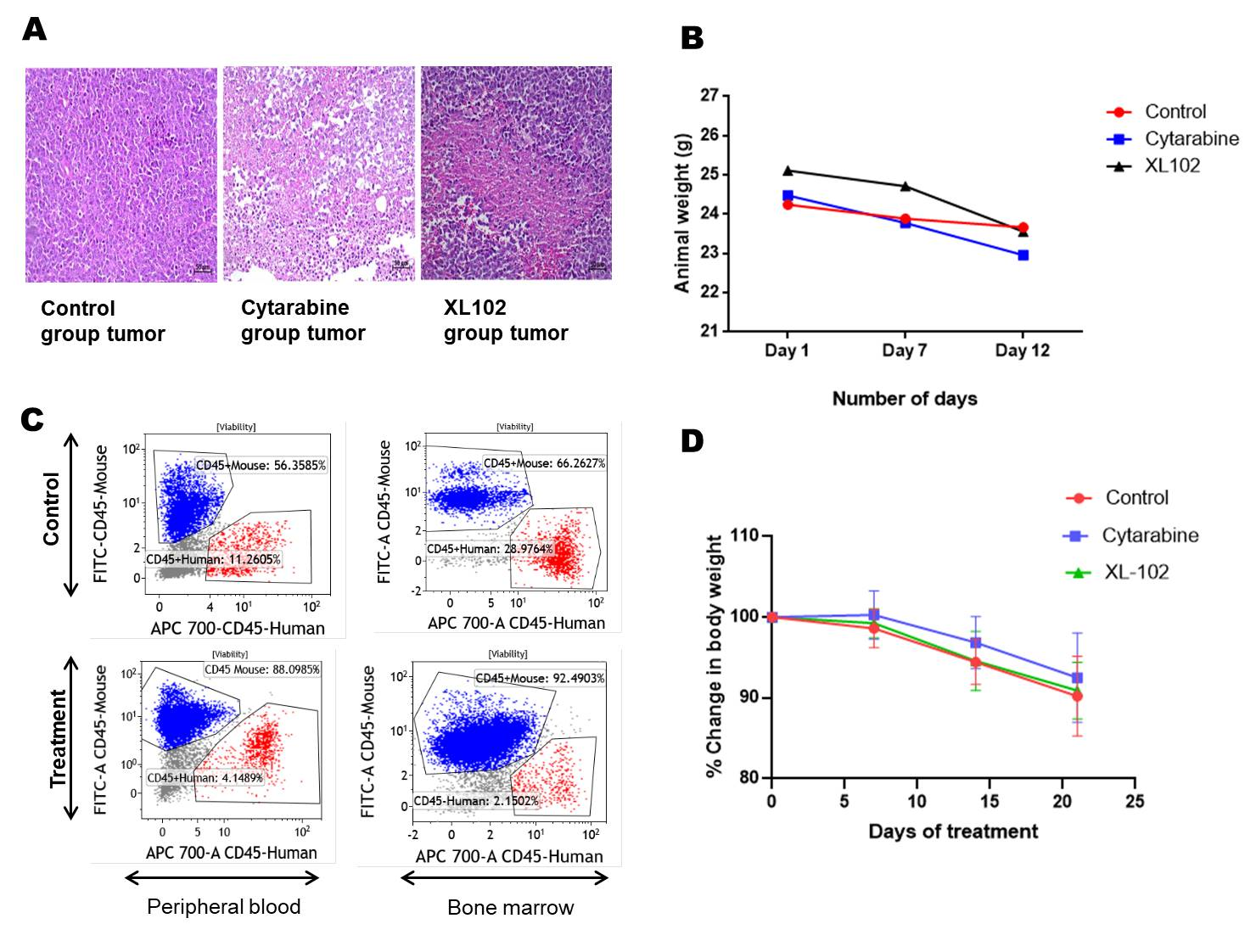


**Supplementary Figure 4: Effect of XL102 treatment on AML animal models** (A) Representative image of H&E-stained tumor sample from each group after 12 days of drug treatment (B) Change in body weight of animals in different groups. (C) Representative image of confirmation of engraftment using dot plot of mCD45 and hCD45 in peripheral blood and bone marrow of animals. (D) Percentage changes in body weight of animals in different groups.


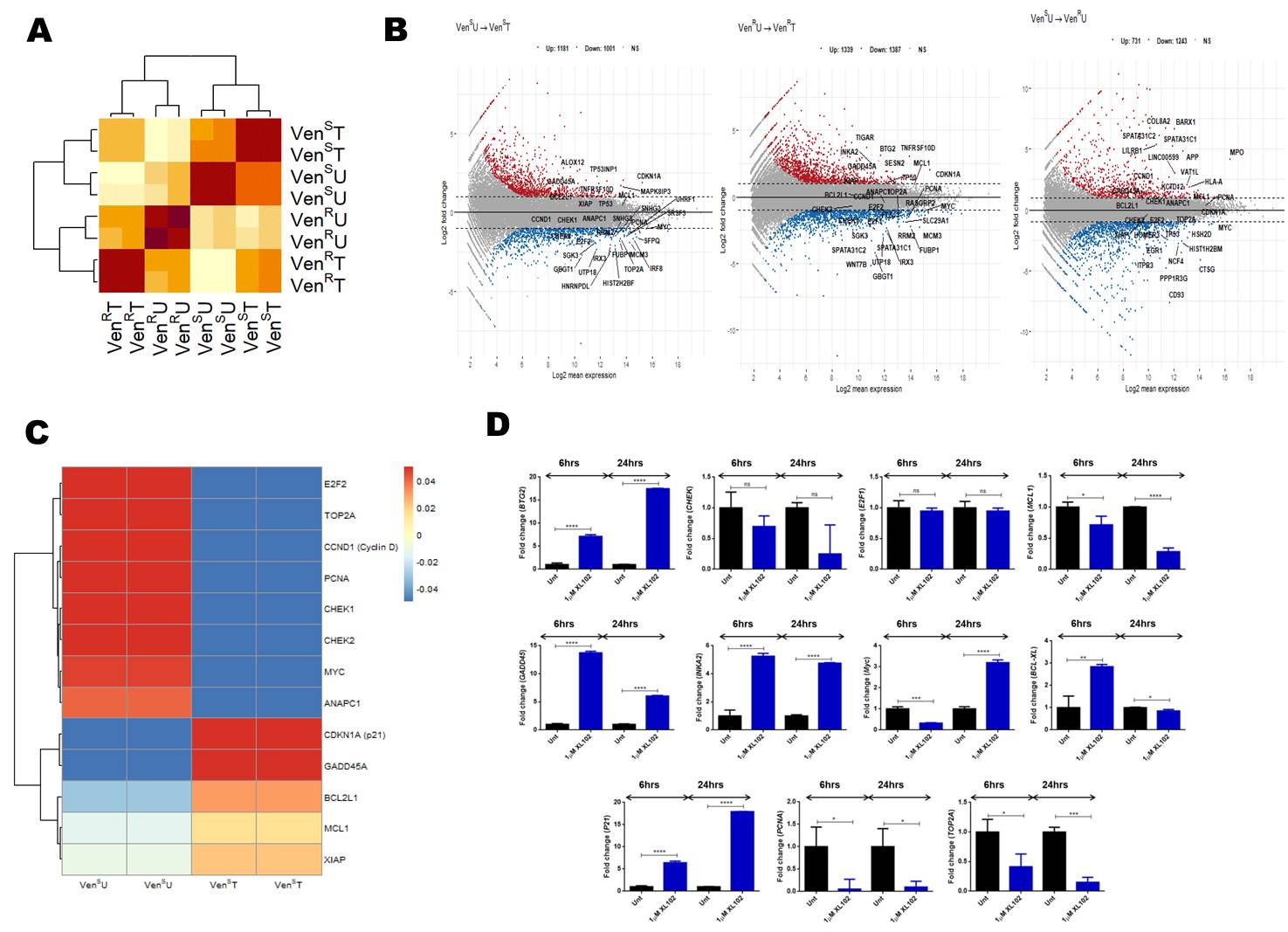

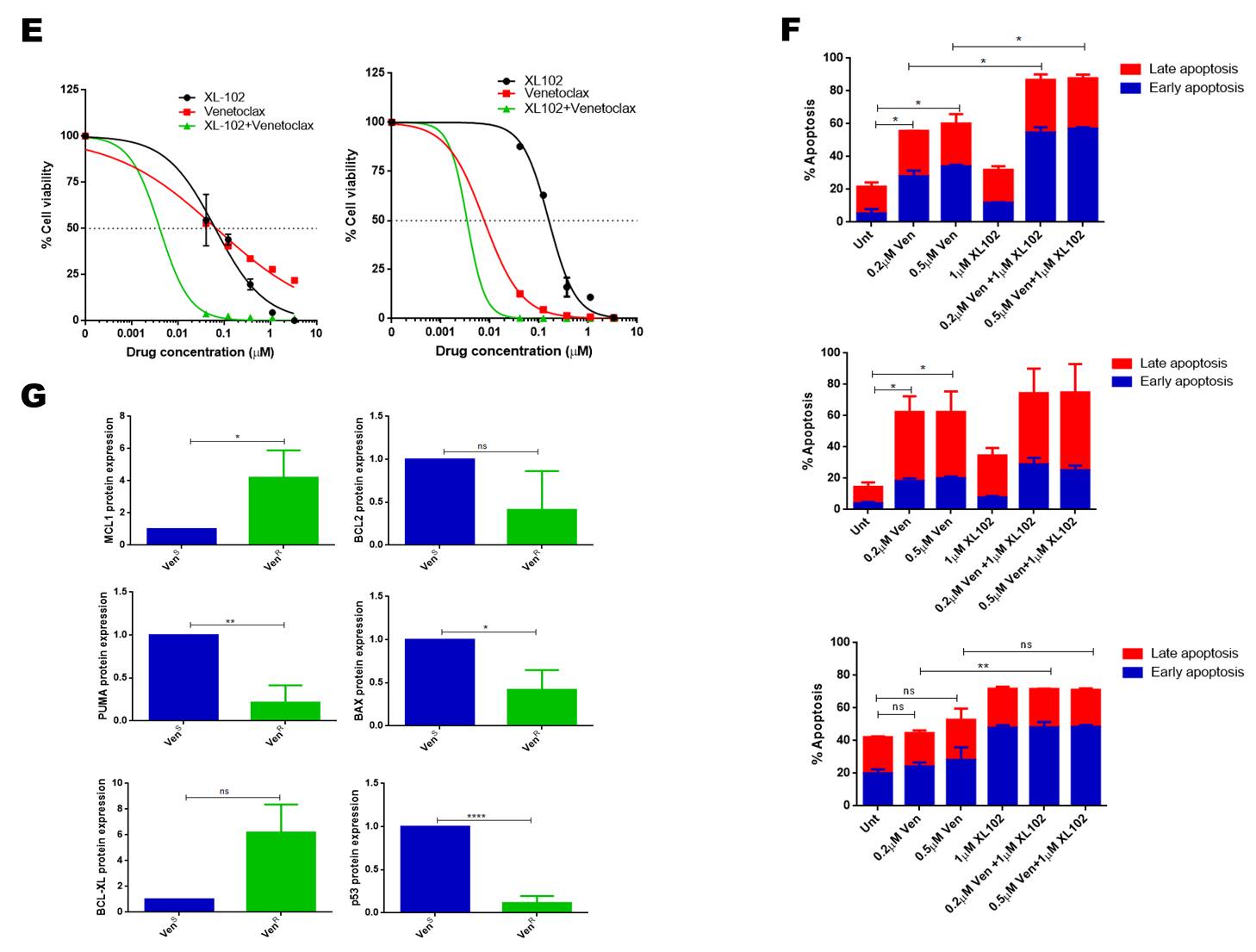


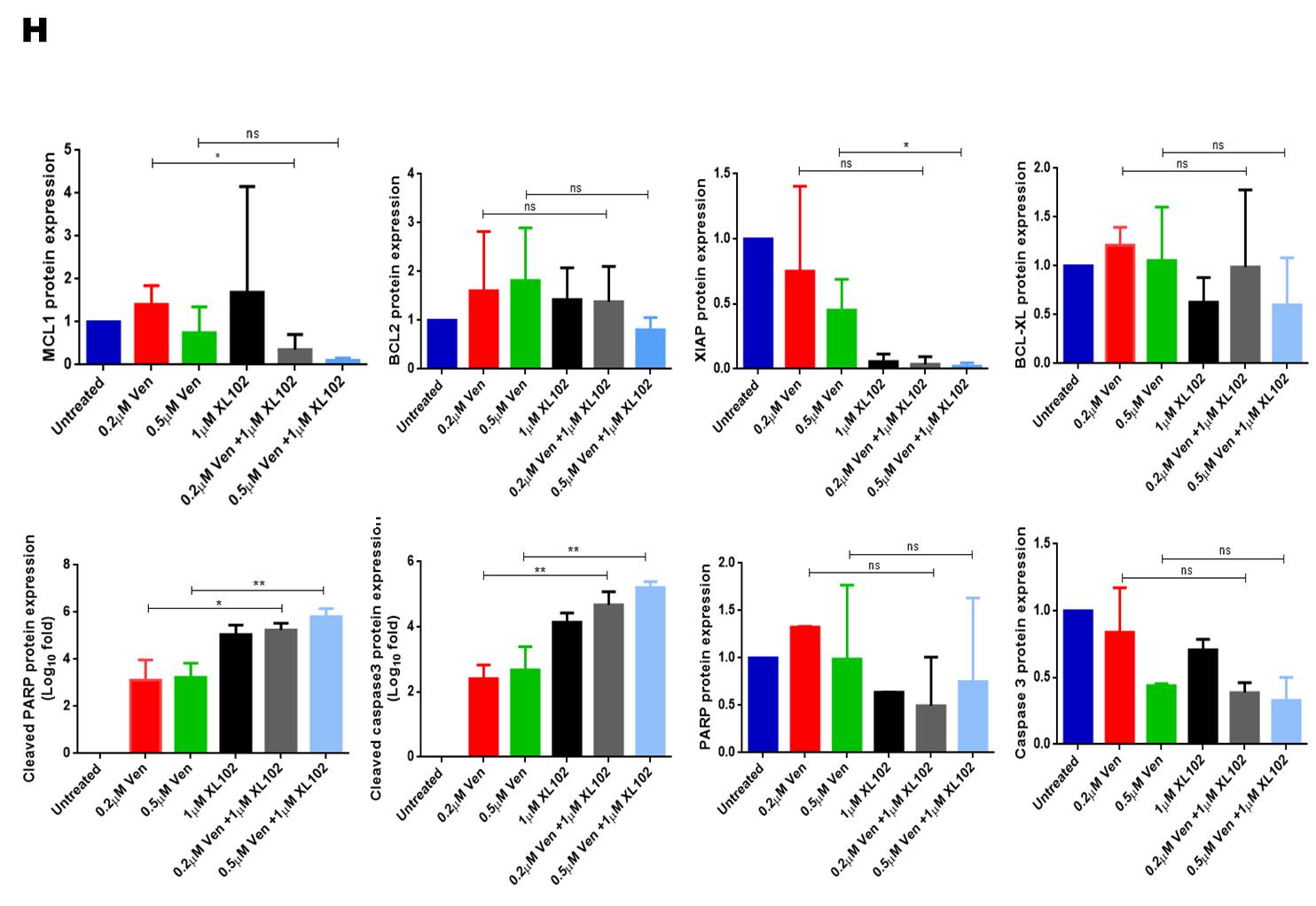


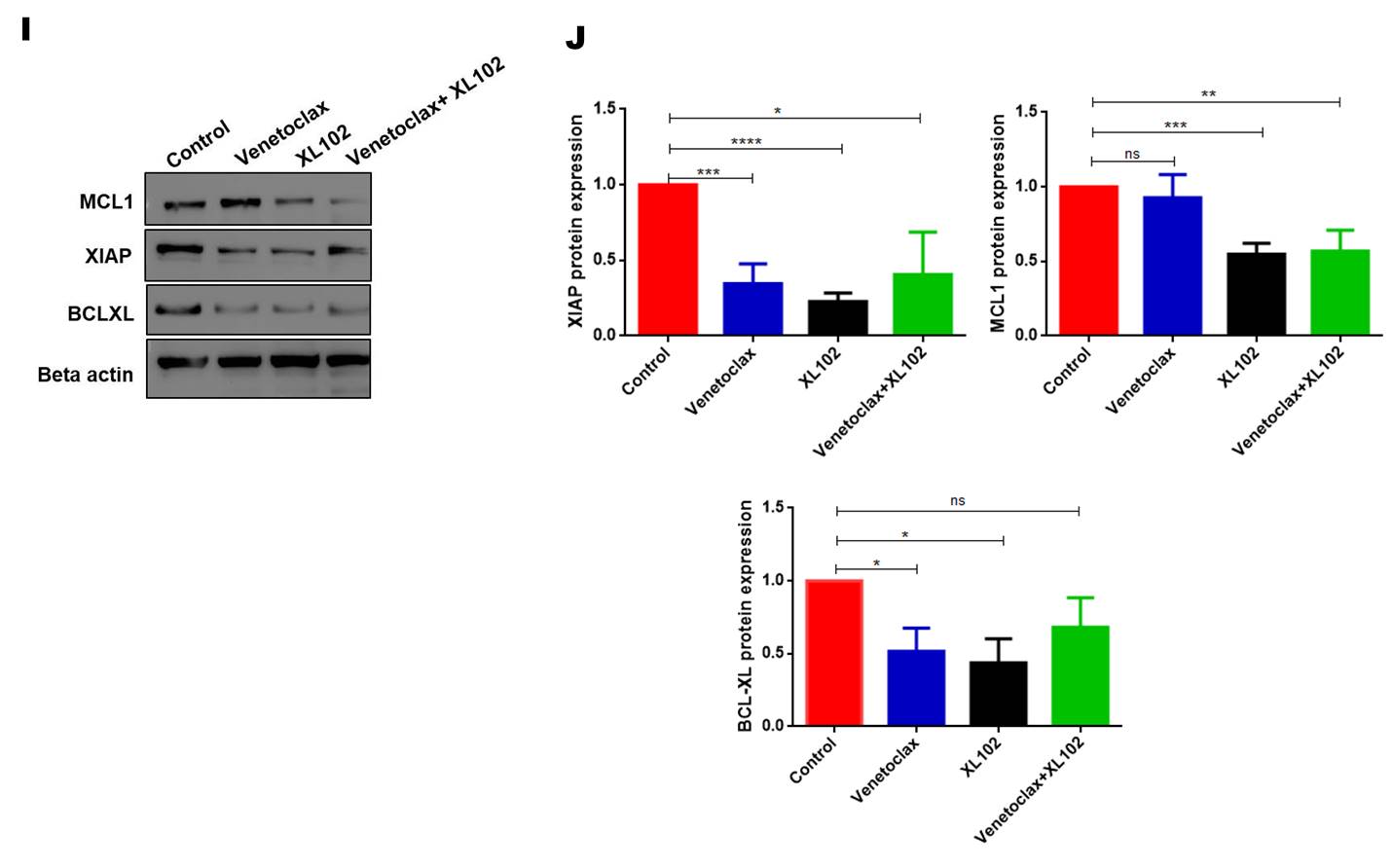


**Supplementary figure 5: Combination of XL102 with Venetoclax shows synergy in AML cells.** (A) Pearson’s correlation matrix based on the gene expression showing the exclusiveness of all four conditions (Parental untreated, parental treated, resistant untreated and resistant treated). There is a higher correlation found between the parental and resistant samples respectively. (B) MA plot representing differential analysis of the three comparisons (Parental untreated vs resistant untreated, parental untreated vs parental treated and resistant untreated vs resistant treated) and highlighting the significantly up or down-regulated genes in each of them. (C) GSEA analysis of Ven^S^ cells showing differentially regulated genes. (D) Q-PCR analysis of modulated genes after XL102 treatment for 24hrs. (E) The combinatorial treatment of XL102 with Venetoclax shows synergy in OCI AML2 cells as the combination index values were less than 1 in all combinations. (F) Enhanced apoptosis was observed after a combination of XL102 and Venetoclax using Annexin/Pi staining in AML cells. (G) Western blot quantification of basal level expression of apoptotic proteins and p53 in MOLM13 Venetoclax sensitive versus resistant cells (H) Quantification of western blots of apoptosis protein after combination of XL102 and Venetoclax in MOLM13 Venetoclax resistant cells. (I-J) Determination of apoptosis markers from tumor excised from xenografts and the quantification of blots.
